# A novel transgenic zebrafish model for blood-brain and blood-retinal barrier development

**DOI:** 10.1186/1471-213X-10-76

**Published:** 2010-07-23

**Authors:** Jing Xie, Eric Farage, Masahiko Sugimoto, Bela Anand-Apte

**Affiliations:** 1 Department of Ophthalmic Research, Cole Eye Institute, Cleveland Clinic, Cleveland, OH, USA; 2 Department of Molecular Medicine, Cleveland Clinic Lerner College of Medicine at Case Western Reserve University, Cleveland, OH, USA

## Abstract

**Background:**

Development and maintenance of the blood-brain and blood-retinal barrier is critical for the homeostasis of brain and retinal tissue. Despite decades of research our knowledge of the formation and maintenance of the blood-brain (BBB) and blood-retinal (BRB) barrier is very limited. We have established an *in vivo *model to study the development and maintenance of these barriers by generating a transgenic zebrafish line that expresses a vitamin D-binding protein fused with enhanced green fluorescent protein (DBP-EGFP) in blood plasma, as an endogenous tracer.

**Results:**

The temporal establishment of the BBB and BRB was examined using this transgenic line and the results were compared with that obtained by injection of fluorescent dyes into the sinus venosus of embryos at various stages of development. We also examined the expression of claudin-5, a component of tight junctions during the first 4 days of development. We observed that the BBB of zebrafish starts to develop by 3 dpf, with expression of claudin-5 in the central arteries preceding it at 2 dpf. The hyaloid vasculature in the zebrafish retina develops a barrier function at 3 dpf, which endows the zebrafish with unique advantages for studying the BRB.

**Conclusion:**

Zebrafish embryos develop BBB and BRB function simultaneously by 3 dpf, which is regulated by tight junction proteins. The *Tg(l-fabp:DBP-EGFP) *zebrafish will have great advantages in studying development and maintenance of the blood-neural barrier, which is a new application for the widely used vertebrate model.

## Background

The central nervous system (CNS) has developed specialized "barriers" to isolate neurons from the blood stream. These barriers are critical for neurological function, as they maintain a stable environment with the regulation of ionic balance and nutrient transport and blockage of potentially toxic molecules. The CNS has two types of barriers: endothelial and epithelial [1,2]. The blood-retinal barrier (BRB) consists of an inner BRB, formed by endothelial cells lining the retinal blood vessels and the outer BRB formed by the retinal pigment epithelium (RPE), a layer of epithelial cells between the retina and non-neuronal choroid [2,3]. The blood-brain barrier (BBB) and the blood-spinal cord barrier are endothelial barriers located within the cerebral vessels of the brain and the spinal cord, whereas the barrier between blood and the cerebrospinal fluid (blood-CSF barrier) is formed by the epithelial cells of the choroid plexus [1,2].

Both the endothelial and epithelial barriers have tight junctions, which seal the intercellular cleft of endothelial or epithelial cells and restrict paracellular diffusion of water-soluble molecules[4]. A number of tight junction proteins have been identified[1,5,6] which include cytoplasmic adapter proteins such as zona occludens-1 (ZO-1), that link trans-membrane proteins such as occludin and claudins to the cytoskeleton. While occludin and claudins are tight-junction-specific, ZO-1 is also a component of adherens junctions[1,5,6]. Studies on these proteins have determined that tight-junctions, initially regarded as static and rigid, are dynamic structures capable of rapid modulation in response to physiological or pathological signals. Claudin 5a has been recently shown to be essential for the establishment of a neuro-epithelial barrier and zebrafish brain ventricular lumen expansion[7].

The dearth of knowledge on BBB/BRB development and disruption is likely due to the fact that BBB/BRB research in the past three decades has been based mainly on *in vitro *models of cultured cells and experiments of *in situ *brain/eye perfusion. An *in vivo *animal model, which can be studied without disrupting the organs, will be critical to address the pathophysiology of BBB/BRB development. Almost all vertebrates, including teleosts, have a BBB with similar functional characteristics [8,9]. The teleost zebrafish (*Danio rerio*) has proven to be a powerful model system to study mechanisms of organogenesis [10], including development of the circulatory system [10,11]. Recently, Jeong et al [12] have examined the BBB in zebrafish. Using molecular markers and injection assays they have demonstrated that a functional endothelial-based BBB is established as early as 3 dpf (days post fertilization) [12].

The characteristics of the BRB, including the molecular and cellular components, development, maintenance and function have not been studied as extensively, but are believed to be very similar to the BBB. Increased vascular permeability and breakdown of the BRB underlies the vision loss in diseases such as retinopathy of prematurity, diabetic retinopathy and age-related macular degeneration [3]. Although a number of studies have suggested a role for tight junction or adherens junction[13] proteins such as occludin[13-15], claudin-5[16], ZO-1[17,18] and VE-cadherin[19-21] in the maintenance of the BRB, our understanding of the molecular mechanisms contributing to the BRB breakdown in pathological conditions is incomplete.

Because of its tissue transparency and rapid development, we hypothesized that the zebrafish would be a good model system to examine the molecular mechanisms regulating the development and maintenance of the BRB. We have determined that the embryos of zebrafish develop a functional BRB in the hyaloid vessels by 3 dpf. We have generated a transgenic zebrafish line that can display the formation, disruption and reconstruction of the BBB and BRB *in vivo*. It will have great advantages in studying the blood-neural barrier through forward-genetic screens and reverse-genetic techniques.

## Results

### The Blood Retinal Barrier (BRB) and Blood Brain Barrier (BBB) is established at 3 dpf in zebrafish

To determine the temporal sequence of establishment of the BRB and BBB in zebrafish, we injected two fluorescent dyes into the circulatory system of *Tg(flk1:mCherry) *[22,23] embryos at 2 dpf, 2.5 dpf and 3 dpf. The vasculature of *flk1:mCherry *embryos is labeled with a red fluorescent protein, mCherry. Two tracers were utilized for this purpose, fluorescein dextran 4 (FD4-4000 Da), to detect large molecule diffusion and sodium fluorescein (376 Da), a small molecule tracer used as a marker of vascular permeability in routine clinical practice.

**Figure 1:**
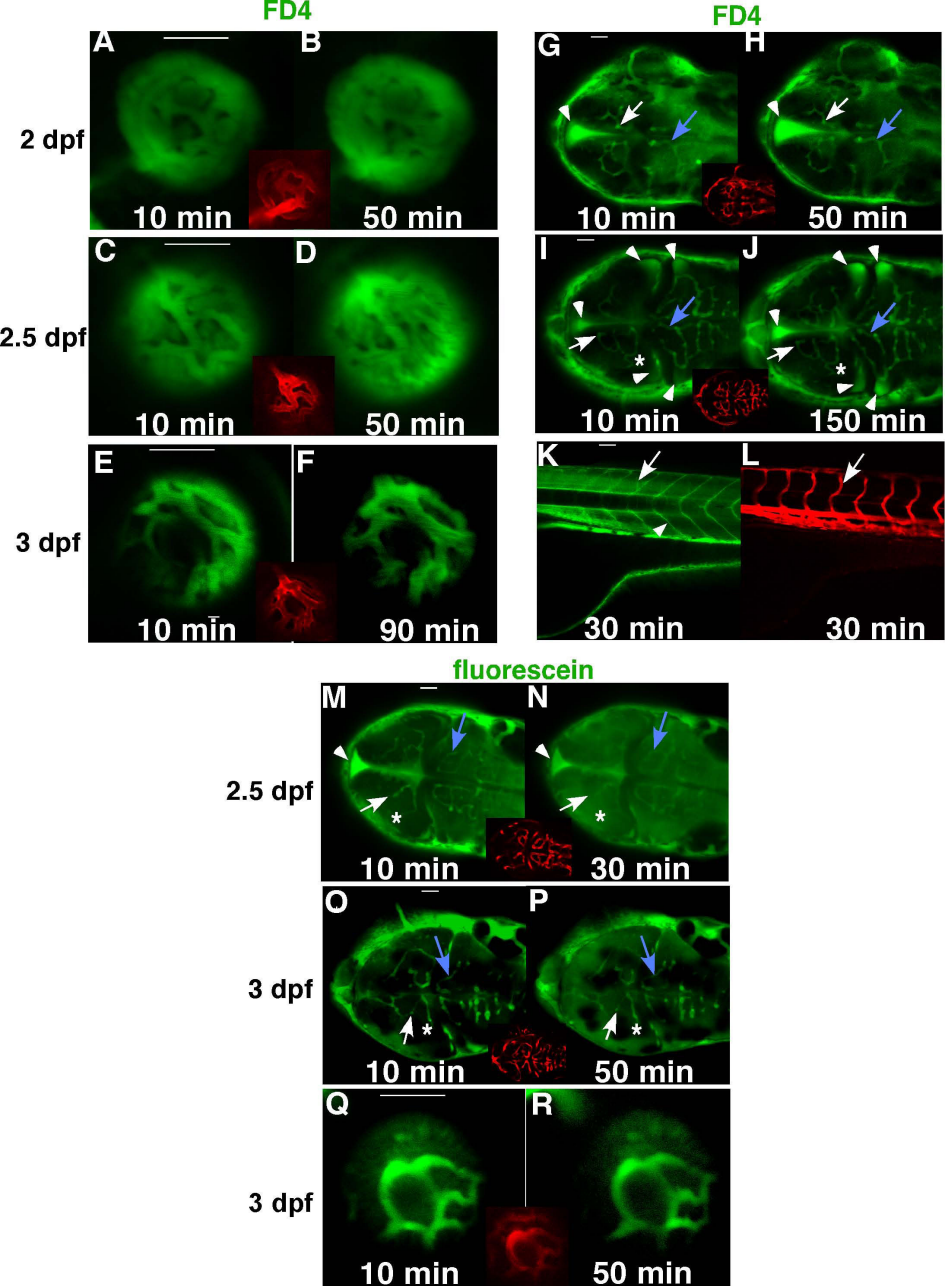
**Development of the BRB and BBB in zebrafish**. FD4 (4,000 Da, green) was injected to the sinus venosus of *Tg(flk1:mCherry) *embryos at 2 dpf (A, B, G, H), 2.5 dpf (C, D, I, J) and 3 dpf (E, F, K. L). Live confocal images of the hyaloid vessels (A-F, side view), brain vessels (G-J, dorsal view) and trunk vessels (K-L, side view) were obtained from 10 to 150 minutes after injection. Insets and panel L are images of established vasculature of the injected embryos (red). In the brain, boundaries of the middle mesencephalic central artery (MMCtA) are indicated by arrows in G-J, cerebellar central artery (CCtA), by blue arrows in G-J and posterior mesencephalic central artery (PMCtA) by asterisks in I&J. Arrows in K&L indicate intersegmental vessels and arrowhead in K shows the myotomal boundaries. A small molecule, fluorescein (376 Da, green), was also used as a tracer in the injection assay (M-R). In the 2.5 dpf embryos (M&N), the PMCtA (asterisk) could not be differentiated, and boundaries of MMCtA (arrows) and CCtA (blue arrows) were enlarged and became blurred 30 minutes after injection. However in the 3 dpf embryos injected with fluorescein (O-R), boundaries of the MMCtA (arrows), PMCtA (asterisk), CCtA (blue arrows) and the hyaloid vessels (Q&R) remained sharp and clear after 50 minutes of injection. The leaked FD4 accumulated mostly in brain ventricles (arrowheads in G-J). In contrast, most of the leaked fluorescein did not accumulated in the brain ventricle (arrowheads in M&N), but evenly diffused throughout the brain (M-P). Scale bars: 50 um.

We observed that the BRB in hyaloid vessels was established in 3-day (3 dpf) embryos, which could retain FD4 in the hyaloid vasculature (Fig. 1E&1F), in contrast to the vessels in 2 and 2.5 dpf embryos, which could not (Fig. 1A-D). Interestingly, FD4 leakage from the trunk vessels of 3 dpf embryos occurred immediately after injection. No FD4 was present in the trunk vessels at 30 minutes after injection, being diffused over the entire trunk with some dye accumulation in the myotomal boundaries (arrowhead in Fig. 1K), and minimal FD4 in the intersegmental vessels (arrows in Fig. 1K&1L). In addition, we examined the permeability of the brain vasculature, particularly the central arteries such as middle mesencephalic central artery (MMCtA), posterior mesencephalic central artery (PMCtA), and cerebellar central artery (CCtA). In the 2dpf and 2.5dpf embryos, although we observed some dye leakage over time with accumulation mostly in the brain ventricles (arrowheads in Fig. 1G-J), the boundaries of the FD4-infused MMCtA (arrows in Fig. 1G-J) and CCtA (blue arrows in Fig. 1G-J) remained sharp and clear at 50 or even 150 minutes after injection, indicating that the endothelial barrier against FD4 is established in the central arteries at 2 dpf.

In contrast to FD4, most of the leaked sodium-fluorescein did not accumulate in the brain ventricles (arrowheads in Fig. 1M&1N), but diffused evenly throughout the entire brain (Fig. 1M-P). In the 2.5 dpf embryos, after 30 minutes of injection, the boundaries of the central arteries could not be differentiated from adjacent brain tissue (arrows, blue arrows and asterisks in Fig. 1M&1N). In 3 dpf embryos, although the diffusing fluorescein caused an increased background throughout the brain, the boundaries of the central arteries remained sharp and clear at 50 minutes after injection (arrows, blue arrows and asterisks in Fig. 1O&1P), suggesting that the endothelial barrier against fluorescein has been established in the vessels. Similar observations were made in the retina, which suggests that the BRB against small molecules is formed in the hyaloid vasculature at 3 dpf as well (Fig. 1Q&1R).

### Claudin-5 is a marker for the CNS vasculature

Claudin-5 is a tight-junction protein expressed in the BBB. We used a monoclonal antibody against the C-terminal region of mouse claudin-5, to examine the spatial expression of claudin-5 in the zebrafish CNS vasculature. The antibody used in these experiments was raised against the 20-residue peptide of the C-terminal region of mouse claudin-5, which has a similar sequence to two zebrafish claudin-5 genes.

**Figure 2:**
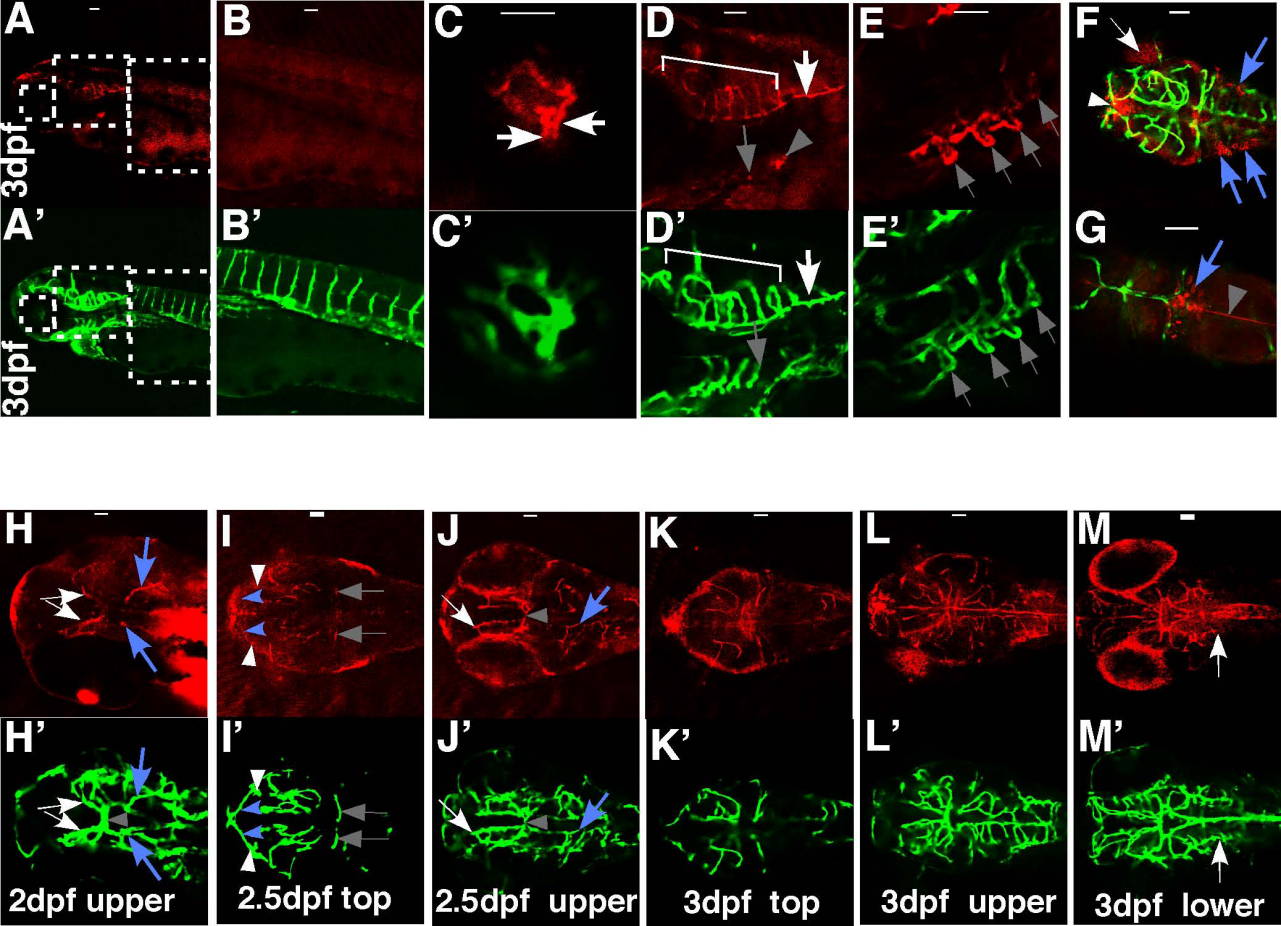
**Spatial and temporal expression of claudin-5 in the developing BBB**. *Tg(flk1:EGFP) *embryos at 2 dpf, 2.5 dpf, and 3 dpf were stained with a monoclonal claudin-5 antibody. Confocal images of whole mount embryos were analyzed for claudin-5 expression (red) (A-E and H-M; Alexa Fluor 568 labeled secondary antibody) in developing blood vessels (green) (A'-E' and H'-M'; EGFP labeled vascular endothelial cells; F&G, merged pictures). All the samples are oriented with anterior to the left. A-E and A'-E', lateral views; other panels, dorsal views. Scale bars: 50 um.

We observed that the claudin-5 signal co-localizes with EGFP expressing endothelial cells of the hyaloid and brain vasculature (Fig. 2C&2D), but not of the trunk vessels (Fig. 2B). The 1st to 4th branchial arches (shaded arrows in Fig. 2E) and the anterior pronephros (shaded arrowhead in Fig. 2D) express claudin. These results demonstrate that the mouse claudin-5 antibody is a good marker for the BBB and BRB of zebrafish. At 3 dpf, claudin-5 immunoreactivity is present in the hyaloid (Fig. 2C&2C') and brain vasculature (Fig. 2D&2D') but not in the trunk vessels (Fig. 2B&2B'). Panels B-D and B'-D' are enlarged views of the dashed squares in A and A'. (Fig. 2D&2D') The claudin-5 antibody binds to the central artery (bracket) and the primordial hindbrain channel (PHBC, arrow). Besides the CNS vasculature, claudin-5 is also present in the 1st to 4th branchial arches (shaded arrows in Fig. 2E&2E') and the anterior pronephros (shaded arrowhead in Fig. 2D) that is localized near the 4th branchial arch (shaded arrow in Fig. 2D). The polygonal RPE cells (arrow) and epithelial cells in the third (arrowhead) and the fourth ventricles (blue arrows) as well as the mid plane of the brain (shaded arrowhead) have a strong claudin-5 signal (Fig. 2F&2G, merged red and green images).

### Expression of claudin-5 in the developing BBB

Temporal expression of claudin-5 was evaluated in the developing BBB. Four pairs of central arteries that develop via angiogenesis, sprout into brain parenchyma as early as 2dpf [24]. At 2 dpf (Fig. 2H&2H'), the MMCtA (arrows) and CCtA (blue arrows), two pairs of newly developed central arteries, express claudin-5 while the basal communicating artery (BCA, shaded arrowhead) that had developed during the earlier vasculogenesis stage does not express claudin-5 (Fig. 2I &2I'; 2J &2J'). Starting at 2.5 dpf, vessels that have developed during the vasculogenesis stage start to express claudin-5. This includes the middle cerebral vein (MCeV) (shaded arrows in Fig. 2I) in the top layer and the BCA (shaded arrowhead in Fig. 2J) in the upper middle layer of the head. The intensity of staining in blood vessels that develop via angiogenesis after 2 dpf such as MMCtA (arrows) and CCtA (blue arrows), increases over time. Like the mesencephalic vein (MsV, arrowheads), all the vessels in the top layer show the claudin-5 signal, although some only have a weak staining, such as the anterior cerebral vein (ACeV, blue arrowheads). (Fig. 2K-M, K'-M'). At 3 dpf, all the brain vessels express claudin-5. The spatial and temporal expression of claudin-5 is consistent with our results with leakage of injected dyes as well as those described previously [12] in that the zebrafish BBB is fully developed by 3 dpf. This result also suggests that claudin-5 expression is a useful marker for the development of the zebrafish BBB.

### Expression of claudin-5 in the developing BRB

**Figure 3:**
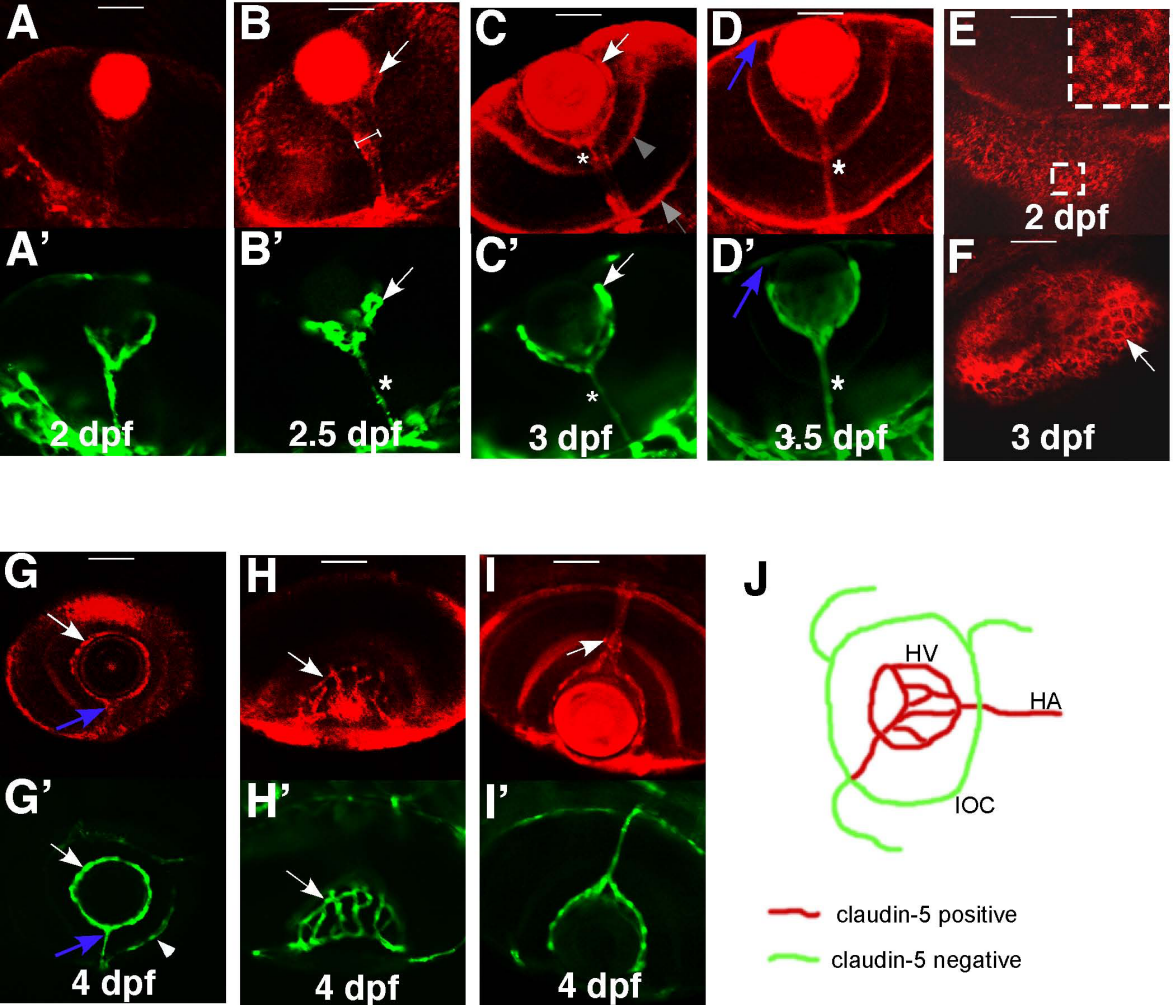
**Spatial and temporal expression of claudin-5 in the developing BRB**. *Tg(flk1:EGFP) *embryos and larvae from 2 dpf to 4 dpf were stained with mouse anti-claudin-5. Confocal images of whole mount were analyzed for claudin-5 (red) (A-I; Alexa Fluor 568) expression in hyaloid blood vessels (green) (A'-D' and G'-I'; EGFP). All the panels are dorsal views except G&G'. The claudin-5 signal in the hyaloid vessels at 2 dpf (A&A') is minimal. At 2.5 dpf (B&B'), and 3 dpf (C&C') the staining in the hyaloid vasculature is increased. Claudin-5 is also expressed in the hyaloid artery (asterisks) and outer limiting membrane of the retina (shaded arrow) and inner plexiform layer (shaded arrowhead). (D&D') At 3.5 dpf, the wider cone-shape staining is lost and the claudin-5 signal overlaps completely with the HA (asterisks). The vessel connecting the HV and the inner optic circle (IOC) also has a strong claudin-5 signal (blue arrows in D&D', G&G'). (E&F) In the retina, the claudin-5 signal does not clearly outline the polygonal RPE (arrow) until 3 dpf. The insert is an enlarged view of the dashed square. At 4 dpf (G-I), the HV (arrows in G&G', H&H') and the HA, but not the choroidal vasculature (such as the IOC, indicated by arrowhead in G'), express claudin-5. The claudin-5 is also expressed around the foramen (opening) through which the HA penetrates the retina (arrow in I). The panel J is a schematic illustrating expression of claudin-5 in optic vasculature of zebrafish. Scale bars: 50 um.

A claudin-5 signal is present at 3 dpf in the hyaloid vasculature of zebrafish (Fig. 2C) consistent with the presence of a vascular barrier. We further analyzed the temporal distribution of claudin-5 protein from 2 to 4 dpf, at which time the BRB is being established (Fig. 1). We found claudin-5 to be present in the net of the hyaloid vasculature (HV; arrows in Fig. 3B&3B') and the hyaloid artery (HA) as early as 2.5 dpf, with a cone shaped localization (H-shape line in Fig. 3B), with a diameter wider than that of the hyaloid artery (asterisk in Fig. 3B'). At 3 dpf, the intensity of the claudin-5 signal is increased in the hyaloid vasculature (arrow in Fig. 3C). At this time claudin-5 signal appears in the inner plexiform layer (shaded arrowhead in Fig. 3C). Claudin-5 in the hyaloid artery appears as a line within the cone-shape staining until 3.5 dpf, after which it overlaps with endothelial cell staining in the hyaloid artery (asterisks in Fig. 3C&3D). At 4 dpf, claudin-5 staining is found at the points of penetration of the hyaloid artery into the retina (arrow in Fig. 3I). Overall, the hyaloid vessels, hyaloid artery and the vessel connecting the hyaloid vessels and the inner optic circle (IOC), but not the IOC itself and other choroidal vessels, express claudin-5 (Fig. 3J). As indicated by the expression of claudin-5 in the RPE cells, the outer BRB is also formed at 3 dpf (Fig. 3E&3F). The expression pattern of claudin-5 in the eye correlated with the observation that the BRB is formed at 3 dpf in zebrafish (Fig. 1).

We also examined the spatial and temporal distribution of ZO-1, a molecular component of both tight junctions and adherens junctions. We observed that ZO-1 was expressed earlier than claudin-5 in the BRB and BBB (see Additional file 1). ZO-1 is not specific to the CNS vasculature, since the ZO-1 antibody binds to a number of endothelial vessels outside of the CNS, such as the intersegmental vessels (see Additional file 1).

### *Claudin-5a and 5b *are expressed in the hyaloid vasculature

**Figure 4:**
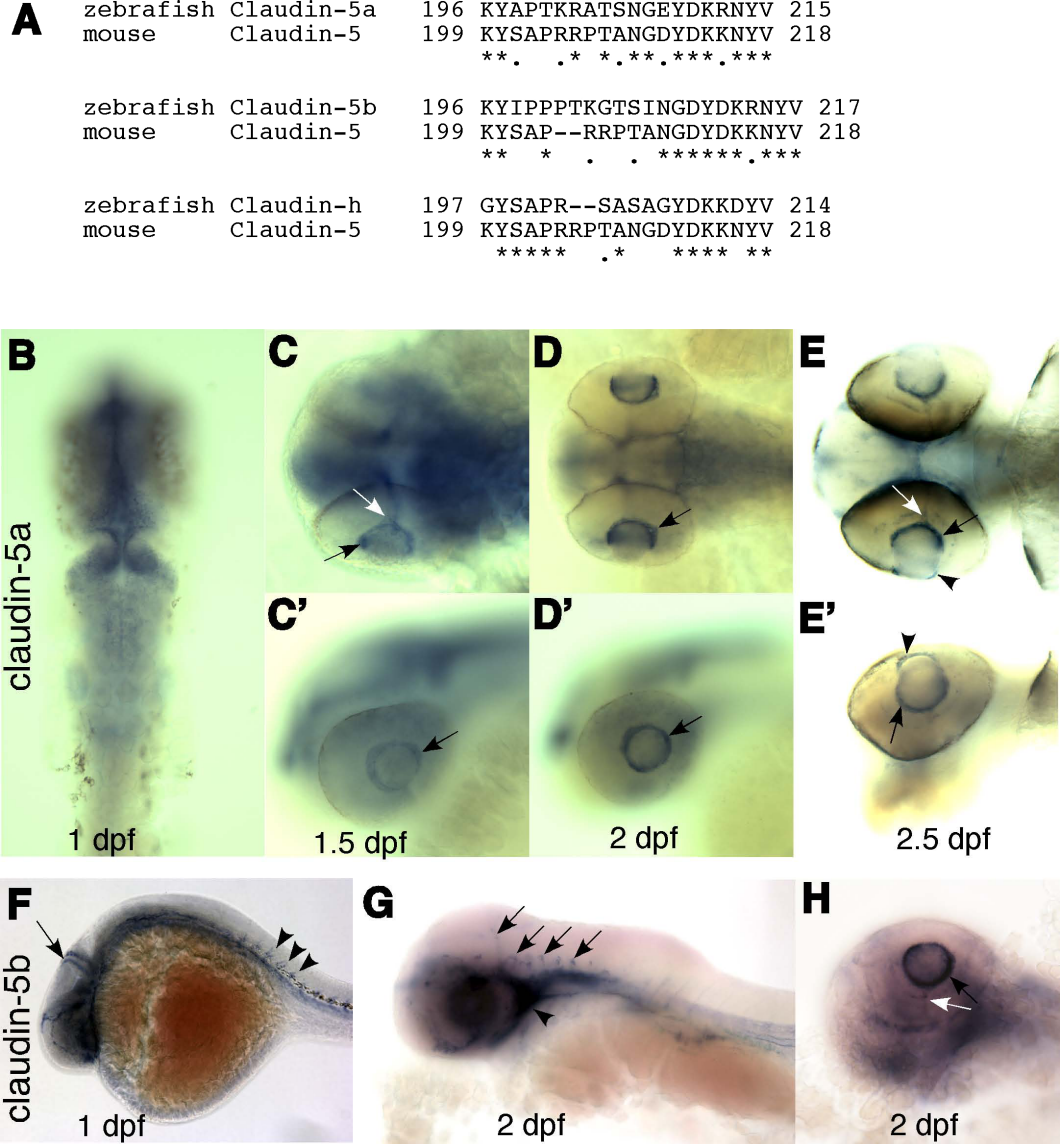
**Claudin-5a and 5b expression in hyaloid vasculature**. (A) The 3' ends of three zebrafish claudin genes have significant homology with the C-terminal of mouse *claudin-5*. Claudin-5a (zgc 85723; GenBank: NM_213274) and claudin-5b (zgc 103419; GenBank: NM_001006044) are mostly homologous to claudin-5a of *Fugu rubripes*, with 82% identical (plus 8% similar) and 75% identical (plus 14% similar) amino acid sequences, respectively. The zebrafish claudin-h (GenBank: NM_131767) is mostly homologous to claudin-3a of Fugu. (B-E') Whole mount *in situ *hybridization of *claudin-5a *expression. (B) *Claudin-5a *is expressed in the CNS (midbrain, hindbrain, ventricular zone, and epiphysis) at 1 dpf. (C-E & C'-E') From 1.5 to 2.5 dpf, claudin-5mRNA is detected in the hyaloid vasculature (black arrows), hyaloid artery (white arrows) and the cornea (arrow heads). (F-H) *Claudin-5b *is expressed in the entire vascular system at 1 dpf (arrow and arrowheads in F), and is confined to the blood vessels of the brain (arrows in G) and cardiovascular system (arrowhead in G) at 2 dpf. Similar to *claudin-5a*, expression of *claudin-5b *in the hyaloid vasculature (black arrow in H) and hyaloid artery (white arrow in H) is seen at 1.5 dpf and lasts till 3 dpf. B-E, dorsal view; C'-E', side view; F-H, side view.

Based on the sequence of the peptide used in generating the mouse claudin-5 antibody, we examined the spatial and temporal expression of five zebrafish claudin genes from 1 to 4 dpf. *Claudin-5a*, *claudin-5b *and *claudin-h *have the highest homology at the C-termini (Fig. 4, A). *Claudin-5a *was expressed in the CNS (midbrain, hindbrain, epiphysis ventricle zones and retina), from 1-2 dpf, which is in accordance with online data (ZFIN ID: ZDB-PUB-040907-1, http://www.zfin.org). *Claudin-5a *mRNA was also present in the hyaloid vasculature from 1.5 to 2.5 dpf (black and white arrows in Fig 4, C-E &4C'-E'). At 3 dpf, it could still be detected in the hyaloid vessels albeit at low levels (data not shown). mRNA of *claudin-5b *was expressed throughout the vasculature system at 1 dpf (Fig 4, F), then gradually lost at 2 dpf, except in the blood vessels of the brain and cardiovascular system (arrows and arrowhead in Fig 4, G). This is in agreement with the gene expression data available through the Zebrafish Information Network (ZFIN ID: ZDB-PUB-040907-1, http://www.zfin.org). Here we extend this study to demonstrate expression of *claudin-5b *in the hyaloid vessels from 1.5 to 3 dpf (Fig 4, H), similar to *claudin-5a*. The partially overlapped expression patterns, as revealed by *in situ *hybridization and immunohistochemistry (Fig. 2, 3 &4), suggest that the anti-mammalian claudin-5 antibody can recognize both claudin-5a and claudin-5b isoforms that constitute the zebrafish BRB and BBB. *In situ *hybridization experiments with zebrafish claudin h gene as well as two negative (sense) controls for cldn 5a and cldn5b showed no expression in the hyaloid vasculature around the lens (see Additional file 2). We have also used a 5.6 kb DNA fragment, upstream of the coding sequence of the claudin-5b to drive expression of EGFP and determined expression in the hyaloid vessels (see Additional file 3). Both these approaches suggest that claudin 5a and 5b are present in the hyaloid vasculature. No expression of the other three zebrafish claudin genes (claudin-h, claudin-k and claudin-1) was detected in the blood vessels of the brain and the hyaloid vessels (data not shown).

### The BRB can be visualized in *Tg(l-fabp:DBP-EGFP) *zebrafish

In order to visualize the blood retinal barrier and blood-brain barrier *in vivo*, we generated transgenic lines of zebrafish that express a vitamin D-binding protein fused with the enhanced green fluorescent protein (DBP-EGFP) under the control of the liver-type fatty acid binding protein (*l-fabp*) promoter. Our goal was to generate a transgenic line that expresses a fusion protein in the plasma that could be used as an endogenous tracer for BBB or BRB breakdown. The l-fabp promoter can drive its expression in hepatocytes from 1.5 dpf to adulthood [25]. DBP is a member of the albumin family and in wild type zebrafish, is translated in the hepatic cells and secreted into the blood circulation. By using the l-fabp promoter and the DBP-EGFP, we generated a transgenic line that expresses the fusion protein in the plasma as an endogenous tracer.

**Figure 5:**
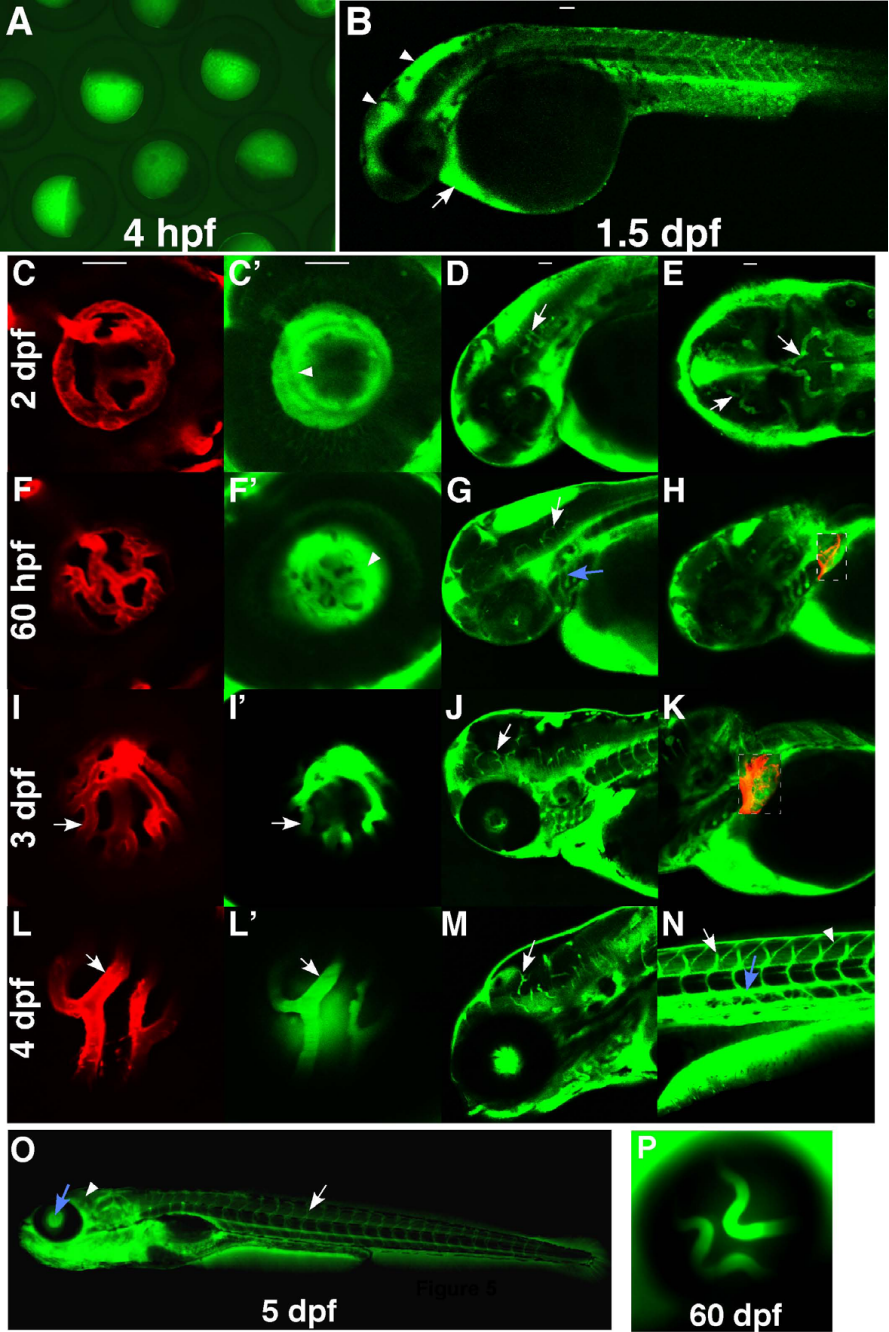
**Expression pattern of DBP-EGFP in the blood plasma of *l-fabp:DBP-EGFP *transgenic zebrafish**. Images were obtained from Tg(*l-fabp:DBP-EGFP;flk1:mCherry) *fish to visualize DBP-EGFP (green) and endothelial cells lining blood vessels (red)(mCherry). The fish were oriented with anterior to the left. All panels are side views except E. (A) green fluorescence in eggs laid by female *Tg(l-fabp:DBP-EGFP) *fish. (B) DBP-EGFP expression at 1.5 dpf. (C, C', D & E) DBP-EGFP expression at 2 dpf. The central arteries (arrows) are the first distinguishable blood vessels. (F, F', G & H) DBP-EGFP expression at 60 hpf. The dashed rectangles in H and K are merged pictures of red and green channels. The branchial arches (blue arrow) can be differentiated at the time, but boundaries of HV are still not clear (arrowhead). At 3 dpf (I, I', J & K), the boundaries of the HV become sharp (arrows in I&I'). More vessels appear in the liver (dashed rectangle) and more brain vessels can be seen (arrows in G and J). At 4 dpf (L, L', M & N), the EGFP-infused hyaloid and brain vessels can be easily differentiated from the fluorescent background (arrows in L, L' and M), as can the intersegmental vessels (arrow in N) and the dorsal aorta (blue arrow). Accumulation of DBP-EGFP in the myotomal boundaries (arrowhead) (N) is observed from leakage out of the vasculature. At 5 dpf (O), the HV (blue arrow) and the trunk vessels (arrow) are distinguishable, but the brain vessels are not (arrowhead), due to the increased fluorescent background in the brain. (P) At 60 dpf, the HV is still distinguishable.

The BBB and BRB in *Tg(l-fabp:DBP-EGFP) *can be displayed from 3 dpf to two months (Fig. 5).

The presence of EGFP fluorescence in all eggs laid by the *l-fabp:DBP-EGFP *transgenic fish (Fig. 5A), indicates that the DBP-EGFP is stored in oocytes as maternal material. At 1.5 dpf, the DBP-EGFP is expressed at low levels and the protein accumulates mostly in the brain ventricles (arrowheads in Fig. 5B) and area of the heart (arrow in Fig. 5B). At 2 dpf, there is a diffuse localization of EGFP in the eye indicating leakage of DBP-EGFP out of the HV (arrowhead in Fig. 5C'). In the brain the EGFP-infused central arteries are the only distinguishable blood vessels (arrows in Fig. 5D&5E). At 60 hpf, blood vessels begin to grow into the liver (Fig. 5H) and the branchial arches (blue arrow in Fig. 5G) become distinguishable, but the boundaries of the HV are not sharp until 3 dpf (Fig. 5F, F', I and 5I'). At 4 dpf, the EGFP-infused hyaloid and brain vessels as well as the intersegmental vessels and the dorsal aorta (arrows and the blue arrow in Fig. 5L-N) can be easily differentiated from the fluorescent background. However the boundaries of the blood vessels in the trunk are not as sharp and distinct (Fig. 5N&5O), as those of the hyaloid and the brain vessels (arrows in Fig. 5L-M). After 5 dpf, the increased fluorescence background in the brain prevents visualization of the brain vasculature (arrowhead in Fig. 5O), but the HV remains distinguishable in the fish up to 60 dpf (Fig. 5P).

### Bradykinin can disrupt the BRB of zebrafish

**Figure 6:**
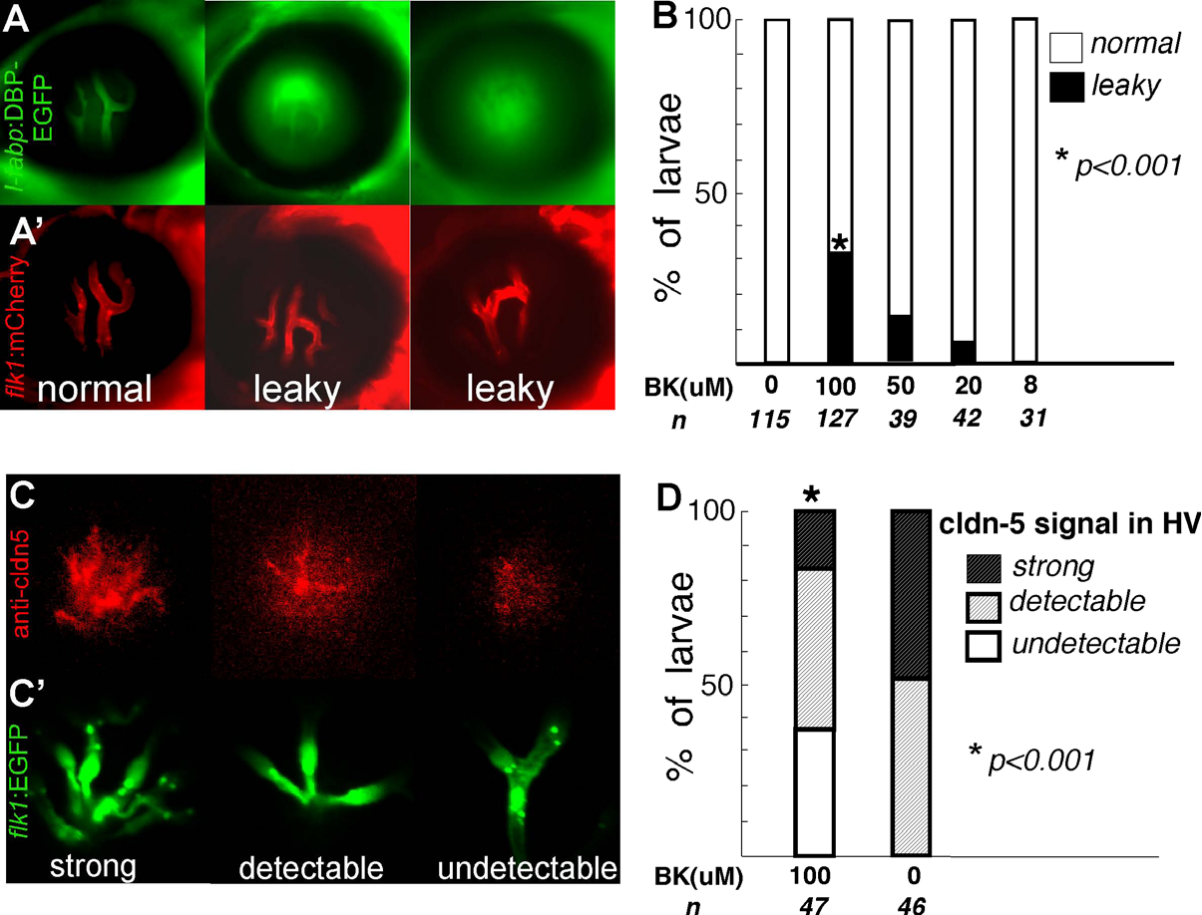
**Bradykinin mediated disruption of zebrafish BRB (A, A' & B)**. The *l-fabp:DBP-EGFP;flk1:mCherry *double transgenic larvae were treated with 8 to 100 μM BK from 5 to 9 dpf. (A) DBP-EGFP (green) in hyaloid vessels of control (left panel), leaky hyaloid vessels (middle and right panel); (A') endothelial lining of blood vessels, mCherry (red). (B) At 9 dpf, the larvae were scored for the presence of leaky hyaloid vessels. Both Fisher's test and chi-square test indicate that the BK treatment results in significantly increased numbers of larvae showing GFP leakage compared with those treated with control buffer (P < 0.001). (C) Claudin-5 expression (red) was evaluated by whole mount immunohistochemistry in *Tg(flk1:EGFP) *larvae exposed to 100 μM BK and scored as strong expression (left panel), detectable (middle panel) and undetectable (right panel); (C') endothelial lining of blood vessels, *flk1:EGFP *(green). (D) Approximately 47 BK treated and 46 untreated *flk1:EGFP *larvae were evaluated for claudin-5 expression in the hyaloid vasculature.

In an effort to determine if *l-fabp:DBP-EGFP *transgenic fish would be a useful tool to screen for inducers and/or inhibitors of the BRB, we examined the ability of bradykinin to induce leakage of EGFP. Bradykinin (BK) is an oligopeptide hormone that can cause BRB breakdown *in vivo *[26]. Extracellular carbonic anhydrase increases retinal vascular permeability through the prekallikrein-kinin pathway[27,28]. Double transgenic *l-fabp:DBP-EGFP;flk1:mCherry *larvae were treated with 8 to 100 μM BK from 5 to 9 dpf. Leaky hyaloid vessels were identified in 31%, 13% and 7% of the 100 μM-, 50 μM- and 20 μM-treated larvae respectively, but in none of the control or 8 μM-treated samples (Fig. 6A,B). In a separate experiment, *flK1:EGFP *larvae were stained with the claudin-5 antibody after treatment with 100 μM BK. Claudin 5 expression was lost in the hyaloid vasculature of 36% of the larvae (Fig. 6 C, D). The apparently normal morphology of the retinal vasculature as visualized by the red fluorescence (*flk:mcherry)*, suggests the absence of generalized vascular pathology with bradykinin which likely has specific effects on the barrier function of the retinal vessels. In addition, direct injection of bradykinin (2 nL of 20 uM bradykinin) into the eyes of *l-fabp:DBP-EGFP;flk1:mCherry *fish resulted in leakage of DBP-EGFP out of the hyaloid vasculature in 90% of injected eyes (n = 20) with no effect in the contralateral control injected eyes (see Additional file 4).

## Discussion

Disruption of the BBB or BRB is a crucial event in the development and progression of a number of diseases. Loss of barrier function leading to increased vascular permeability in the brain or retina can be a cause or consequence of the pathology. A detailed understanding of the physiological processes involved in the development and maintenance of the blood-neural barrier is critical for the identification of therapeutic targets. One of the major limitations in this regard has been the absence of an easily regulated *in vivo *system that allows alterations of these barriers. The BBB and BRB, initially regarded as static and rigid, have now been proven to be dynamic structures with both paracellular and transcellular pathways capable of rapid modulation in response to physiological or pathological signals [1,29].

Development of the vascular system in the brain can be classified into three phases: vasculogenesis, angiogenesis and BBB formation. During brain angiogenesis, sprouting vessels from the perineural plexus grow into the proliferating neuroectoderm and form a capillary network. At this time, the vessels appear to be permeable to small molecules [30-33] but not to plasma proteins [31,34]. Although there have been a large number of studies examining the molecular mechanisms involved in vasculogenesis and angiogenesis, very limited information is available for the development of the BBB [35-38]. The Src-suppressed C kinase substrate is a factor induced by high oxygen tension. Its expression in brain astrocytes leads to decreased vascular endothelial growth factor (VEGF) and increased angiopoietin-1 secretion, which have been suggested to be important for the cessation of brain angiogenesis and formation of the BBB [36]. More recent reports demonstrate that Wnt signaling is required for CNS angiogenesis [35] and may play a role in the initiation of the development of the BBB in mice [37,38]. Hypoxia has been shown to regulate the barrier function of neural blood vessels by reducing the expression of claudin 5 in endothelial cells [16]. In addition, claudin-5 deficient mice show a size selective (<800 Da) loosening of the blood-brain barrier[39].

In this report we describe *l-fabp:DBP-EGFP *transgenic zebrafish, in which the BBB and BRB can be visualized. This model will be a critical tool for future studies related to the study of blood-neural barrier development and differentiation. The molecular weight of the DBP-EGFP fusion protein, both calculated by the MacVector software and estimated by Western blot data (see Additional file 5), is 78 kDa. Since the zebrafish claudin-5a and claudin-5b are sufficient to block paracellular transport of any molecule of 4000 Da or larger, but not sufficient to block the passage of smaller molecules like sodium fluorescein (376 Da) it is likely that DBP-EGFP would be comparable to high MW compounds.

Human albumin (67 kDa) has been used as an endogenous tracer for blood-brain barrier studies and as an indicator of compromised BBB function in a number of pathophysiological conditions. Under physiological conditions albumin crosses endothelial cell wall via transcytosis. However, paracellular transport of albumin in various sized microvessels has been observed under inflammatory conditions and following treatment with reagents that affect the integrity of tight-junctions. The zebrafish DBP is a member of albumin family and is highly homologous to the human DBP and thus a good marker to evaluate the integrity of the BBB and BRB. We have recently demonstrated that knock-down of claudin-5a results in the leakage of DBP-EGFP out of the hyaloid vessels (see Additional file 6). These results taken together with the results in Fig. 6, suggest that DBP-EGFP, as an endogenous tracer, may be useful to evaluate the breakdown of the BRB against high molecular weight compounds, either due to developmental defects or pathological conditions.

Although the structures of the BBB and BRB in vertebrates have been fairly well characterized [1-6], information about their development during embryogenesis and their maintenance in adults is limited. Similarities between them suggest that some of the developmental and regulatory mechanisms involved in these two barrier systems may overlap. Many CNS disorders, such as brain tumors, stroke, trauma, multiple sclerosis and neurodegenerative diseases, are associated with a dysfunction of the BBB[1,3]. On the other hand, the presence of the BBB presents a major challenge for delivery of therapeutic compounds to the brain, as most drugs do not cross the BBB [2,40].

**Figure 7:**
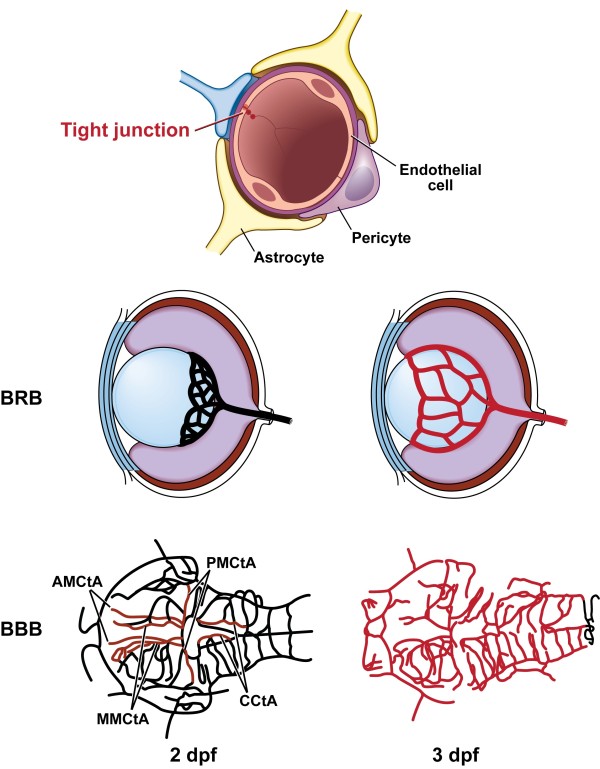
**Schematic illustration of endothelial tight junctions and claudin-5 expression in retinal and brain vasculature of zebrafish embryos at 2 dpf and 3 dpf**. Four pairs of the central artery express claudin-5 at 2 dpf (red). The figure was adapted from the online data of Dr. Brant Weinstein's lab (zfish.nichd.nih.gov/zfatlas). AMCtA: anterior mesencephalic central artery; MMCtA: middle mesencephalic central artery; PMCtA: posterior mesencephalic central artery; CCtA: cerebellar central artery.

In the present study we used FD4 as a tracer of large molecular weight and fluorescein as a small molecular weight tracer. In zebrafish, FD4 (4,000 Da) but not fluorecein (376 Da) could be retained in the vasculature at 2 dpf when the central arteries started to express claudin-5. This suggests that claudin-5 is likely to be critical for the barrier against large molecules and some additional junction protein may be needed for the barrier against small molecules. Our results with tracer injections, the claudin-5 antibody staining, as well as the observations on the *l-fabp:DBP-EGFP *embryos, revealed a subtle difference between the formation of BRB and BBB. The BRB against both large and small molecules developed gradually in the hyaloid vessels and was formed by 3 dpf, similar to the BBB against small molecules. In contrast the BBB against large molecules is formed in the central arteries at 2 dpf (Fig. 7), and in other blood vessels at 2.5 dpf. The developmental process of the zebrafish BBB is similar to results obtained from other previously studied vertebrates, in that the brain vessels of the angiogenesis stage are permeable to small molecules but not to plasma proteins [31,34].

However, not all cerebral blood vessels are impermeable in adult zebrafish. The blood vessels of the circumventriclar organ lack a BBB structure [12,41], as these vessels have special physiological functions. Consistent with this, the accumulation of EGFP in the brain of the *l-fabp:DBP-EGFP *larvae results in a high fluorescent background and the brain vasculature becomes indistinguishable from 5 dpf onward. Thus the *l-fabp:DBP-EGFP *will need to be modified to allow conditional expression of DBP-EGFP for studies on the BBB of zebrafish post 5 dpf. In contrast, the zebrafish BRB is easier to evaluate as all the hyaloid vessels develop an inner BRB structure at 3 dpf; the outer BRB also matures at 3 dpf and further seals off the eyes. This is an added advantage in studying the BRB as the hyaloid/retinal vessels can be observed in the transgenic *l-fabp:DBP-EGFP *zebrafish up to 60 dpf.

The zebrafish has been widely used to study eye development and disease [42], but the BRB has not been studied in this model. Alvarez et al [43] have recently examined the morphology and development of the hyaloid and retinal vasculature in zebrafish and identified an important distinction between mammals and zebrafish. In mammals, the regression of the hyaloid vasculature by apoptosis and the formation of the retinal vasculature by angiogenesis are synchronized processes, while in the zebrafish, the hyaloid vessels develop into retinal vessels without regression. In addition, adult zebrafish have tight-junctions in retinal endothelial cells and pericytes along the retinal vessels[44].

Bradykinin is an oligopeptide hormone derived from proteolysis of kininogen and is involved in smooth muscle contraction and relaxation, increased vascular permeability, activation of pain sensory fibers, hypotension and inflammation [45]. Bradykinin activates two specific membrane receptors, B1 and B2 and plays a direct role in diabetes-induced breakdown of the BRB [26-28]. The kininogen and two BK receptors have been identified in zebrafish and two species of the pufferfish [46] and shown to be localized to the brain and eye [47]. The structure of BK is evolutionarily conserved between zebrafish and mouse with only two out of ten amino acids being substituted [48]. Previous studies have determined that mammalian BK has 16% potency of zebrafish BK *in vitro *assays [48]. Although the zebrafish BK receptor B1 is thought to have a ligand-interaction profile distinct from mammalian BK receptors [48], we observed that 31% of the zebrafish larvae treated with 100 μM mammalian BK for four days demonstrated leaky hyaloid vessels. These findings lend credence to the hypothesis that the regulatory mechanisms of BRB may be conserved between zebrafish and humans.

The use of zebrafish as a model organism to study the BBB and BRB has a number of advantages. The ability to carry out forward-genetic screens in zebrafish is one of the models most attractive features. A forward-genetic approach following mutagenesis of *l-fabp:DBP-EGFP *transgenic zebrafish, and screening for a leaky BRB phenotype will identify and characterize zebrafish mutants that affect the establishment and maintenance of the BRB. This phenotype-driven genome-wide screen, which makes no assumptions about the genes involved in the biological processes of interest, can likely reveal novel genetic pathways involved in the development of BRB.

## Conclusions

We have demonstrated that zebrafish have a BBB and BRB structure which is formed at 3 dpf. A transgenic zebrafish line, as well as a monoclonal claudin-5 antibody, can display the formation, disruption and reconstruction of the BBB and BRB. The *l-fabp:DBP-EGFP *transgenic zebrafish will have great advantages in identifying the genes involved in development and maintenance of the BRB, through reverse-genetic techniques and forward-genetic screens. Disruption of BRB by bradykinin demonstrates that the transgenic zebrafish could also be used for experimental testing of therapeutic agents that could potentially be effective in the treatment of retinal or brain vascular leakage.

## Methods

### Zebrafish maintenance and strains

All Zebrafish *(Danio rerio) *studies were conducted in accordance with the Animal Care and use Committee guidelines of the Cleveland Clinic (ARC 08498). Zebrafish were maintained at 28.5°C on a 14-hour light/10-hour dark cycle according to standard procedures [49]. Embryos were obtained from natural spawning and raised at 28.5°C. *Tg(flk1:EGFP) *and *Tg(flk1:mCherry) *lines were a generous gift from the laboratory of Dr. Stainer [23]. Some embryos were treated with 0.1 mM 1-phenyl-2-thiourea (PTU, Sigma P5272) to inhibit pigment formation. No difference in experimental results was observed between PTU-treated and untreated embryos.

### Leakage assay by FD4 and fluorescein injections

FD4 (Sigma, 4,000 Da) and fluorescein sodium (Sigma F6337, 376 Da) were dissolved in embryo medium[49] to final concentrations of 2 mg/ml and 0.1 mg/ml respectively. *Tg(flk1:mCherry) *embryos were anesthetized with 0.2 mg/ml Tricaine (Sigma A5040) at 2 dpf, 60 hpf (hours post fertilization) or 3 dpf. About 3 nl of FD4 or fluorescein were injected to the sinus venosus. Following injection, the embryos were mounted in 1% low-melting agarose and images were taken from 10 to 90 minutes post injection with a confocal microscope (Leica TCS-SP2).

### Whole-mount immunohistochemical staining

*Tg(flk1:EGFP) *embryos or larvae were fixed with 4% paraformaldehyde for 3 hours at 4°C. After washes in 1× PBS, the samples were digested with 0.125% Trypsin (Invitrogen) for 11 to 16 minutes at room temperature, depending on the developmental stages. The samples were incubated in a blocking solution (1% BSA/3% normal goat serum/0.4% Triton X-100/1× PBS) with mouse anti-claudin-5 (Zymed 18-7364, 1:2000) or rabbit anti-ZO-1 (Zymed 61-7300, 1:4000) at 4°C for 8 hours. After thorough washes with 0.4% Triton X-100/1× PBS, the samples were incubated in the blocking solution with the appropriate secondary antibody, Alexa Fluor 568 goat anti-mouse IgG (Invitrogen, 1:2500) or Alexa Fluor 568 goat anti-rabbit IgG (Invitrogen, 1:3000), at room temperature for 1 hour. Confocal mages were taken with the samples mounted in 1% low-melting agarose (Leica TCS-SP2). The vascular nomenclature is labeled according to Isogai et al. [24].

### Whole-mount in situ hybridization

The coding sequences of zebrafish claudin-5a (648bp; GenBank: NM_213274) and claudin-5b (654 bp; GenBank: NM_001006044) were cloned into pCRII-TOPO and the resulting plasmids were linearized with BamHI or XhoI for synthesis of antisense or sense probes. Whole-mount *in situ *hybridization was performed using digoxigenin (DIG)-labeled RNA probes and anti-DIG alkaline phosphatase conjugated antibody as previously described [50]. Transcription of three other zebrafish claudins, claudin-h (GenBank: NM_131767), claudin-k (GenBank: NM_001003464) and claudin-i (GenBank: NM_131770), were also tested from 1 to 4 dpf.

### Generation of *Tg(l-fabp:DBP-EGFP) *fish

The *Tol2 *transposon system [51,52] was used for transgenesis. A 3.5 kb promoter sequence of the *l-fabp *gene [25] was amplified from genomic DNA of wild-type zebrafish. The promoter was inserted into the *Apa*I and *Bam*HI sites of the pT2KXIGΔin vector [52] to replace the EF-1α promoter. The coding sequence of zebrafish DBP was amplified from a cDNA clone (GenBank: BC076230) and inserted into the *Bam*HI of pT2KXIGΔin to make an inframe fusion at the N terminus of EGFP. Transposase RNA was transcribed *in vitro *from the pCS-TP vector. Approximately 1 nl of an injection solution, containing 25 ng/μl circular plasmid DNA and 25 ng/μl transposase RNA, was microinjected into 1- to 2-cell stage embryos as described [52]. EGFP expression was examined by fluorescent microscopy (Nikon EFD-3); embryos with expected expression patterns were raised to establish the transgenic lines.

### Confocal imaging

Live embryos/larvae or immunohistochemical samples were mounted in 1% low-melting agarose. Confocal images were acquired using a Leica DM IRBE inverted microscope coupled to the Leica TCS-SP2 system using a Plan-Apochromat 10×/0.40 lens. Green (for EGFP, FD4 and fluorescein) and red (for mChery and Alexa Fluor 568) channels were excited using an Agron/Krypton and Helium/Neon laser, and emissions were detected using filters set by the Leica Confocal Software.

### Disrupting the BRB with bradykinin

*Tg(l-fabp:DBP-EGFP) *and *Tg(flk1:mCherry) *fish were crossed to obtain embryos bearing double transgenes. From 5 dpf, the larvae were bathed in embryo medium [49] containing 0, 8, 20, 50 or 100 μM bradykinin. At 9 dpf, the larvae were mounted in 2% methylcellulose; leakage of the DBP-EGFP out of the hyaloid vessels was examined by fluorescent microscopy (Nikon EFD-3). *Tg(flk1:EGFP) *larvae were also immunohistochemically stained with anti-claudin-5 after treatment with 100 μM bradykinin.

## Authors' contributions

JX designed and performed research, analyzed and interpreted data and drafted the manuscript. EF and MS performed research. BA-A designed research, analyzed data and wrote the manuscript. None of the authors have any conflict of interest. All authors have read and approved the final manuscript.

## Supplementary Material

Additional file 1 **Expression of ZO-1 in the developing BRB and BBB of zebrafish**. *Tg(flk1:EGFP) *embryos at 2 dpf to 3 dpf were stained with rabbit anti-ZO-1; confocal images were analyzed for ZO-1 expression (red) (A-G; Alexa Fluor 568) and blood vessels (green) (A'-E'; EGFP). A&A', lateral views; the other panels, dorsal views. (A&A') The ZO-1 signal is high in the gut (shaded arrow) and low in the intersegmental vessels (arrows). (B&B') At 2 dpf, ZO-1 is localized to the HV (arrows) and the HA (arrowheads). (C&C') At 3 dpf, the HV (arrows), inner plexiform layer (shaded arrowhead) as well as the outer limiting membrane (shaded arrow) show a strong signal of ZO-1. (D&D', E&E') At 2 dpf, most brain vessels express ZO-1, including the BCA (arrowheads). Similar to the claudin-5 antibody, the ZO-1 antibody binds to many non-endothelial structures in the brain (shaded arrows and shaded arrowhead) besides the brain vasculature (arrows). (F&G) At 2 dpf, the ZO-1 antibody can stain the polygonal RPE cells (arrows) clearly. The insert in G is an enlarged view of the dashed square. Scale bars: 50 um. Click here for file

Additional file 2 ***In situ *hybridization of cldn-5a, cldn-5b and cldn-h**. Wild type embryos at 2.5 dpf were probed with anti-sense probes of cldn5a, cldn5b and cldn5h. The cldn-5a and cldn-5b had very strong signal in the hyaloid vasculature around the lens, while the cldn-h did not. Two sense controls did not show any signal. Click here for file

Additional file 3 **Claudin 5b is expressed in hyaloid vessels**. A 5.6 kb upstream sequence of claudin-5b can drive expression of EGFP in the hyaloid vessels. The inset is an enlarged view of the green hyaloid vessels. Click here for file

Additional file 4 **Bradykinin induces BRB breakdown**. At 5 dpf, 20 double transgenic (*l-fabp:DBP-EGFP;flk1:mCherry*) larvae were injected with 2 uL of 20 uM bradykinin in the right eye (B, B', C and C'). 3 hours after injection, the BRB was found to be disrupted in 18 injected eyes, as indicated by the leakage of DBP-EGFP (green) (B, C) from the hyaloid vessels (red) (B'C') which showed normal morphology. No leakage was observed in any of the BSA injected contra-lateral negative control eyes (A, A'). Click here for file

Additional file 5 **DBP-EFGP is a 78 kDa protein in *l-fabp:DBP-EGFP *zebrafish**. The expression level and molecular weight of DBP-EGFP was analyzed by western blot analysis of lysates from *l-fabp:DBP-EGFP *(A) and wild type (B) embryos using anti-GFP antibodies (Abcam, ab290). A 78 kDa protein was detected in lysates from transgenic embryos. Click here for file

Additional file 6 **Morpholino knockdown of claudin 5a in zebrafish embryos results in BRB breakdown**. Cldn-5a was knocked down by injecting morpholino-cldn5a into double transgenic *l-fabp:DBP-EGFP;flk1:mCherry *embryos at the 2-cell stage. At 4 dpf, leakage of DBP-EGFP(green) from the hyaloid vasculature (red) was observed. A 5 bp-mismatched Morpholino was injected as control and showed an intact BRB. Click here for file

## References

[B1] Abbott NJ Ronnback L Hansson E Astrocyte-endothelial interactions at the blood-brain barrier Nat Rev Neurosci 2006 7 41 53 10.1038/nrn1824 16371949

[B2] Neuwelt E Abbott NJ Abrey L Banks WA Blakley B Davis T Engelhardt B Grammas P Nedergaard M Nutt J Strategies to advance translational research into brain barriers Lancet Neurol 2008 7 84 96 10.1016/S1474-4422(07)70326-5 18093565

[B3] Erickson KK Sundstrom JM Antonetti DA Vascular permeability in ocular disease and the role of tight junctions Angiogenesis 2007 10 103 17 10.1007/s10456-007-9067-z 17340211

[B4] Dejana E Tournier-Lasserve E Weinstein BM The control of vascular integrity by endothelial cell junctions: molecular basis and pathological implications Dev Cell 2009 16 209 21 10.1016/j.devcel.2009.01.004 19217423

[B5] Hawkins BT Davis TP The blood-brain barrier/neurovascular unit in health and disease Pharmacol Rev 2005 57 173 85 10.1124/pr.57.2.4 15914466

[B6] Zlokovic BV The blood-brain barrier in health and chronic neurodegenerative disorders Neuron 2008 57 178 201 10.1016/j.neuron.2008.01.003 18215617

[B7] Zhang J Piontek J Wolburg H Piehl C Liss M Otten C Christ A Willnow TE Blasig IE Abdelilah-Seyfried S Establishment of a neuroepithelial barrier by Claudin5a is essential for zebrafish brain ventricular lumen expansion Proc Natl Acad Sci USA 107 1425 30 10.1073/pnas.0911996107 20080584 PMC2824400

[B8] Bundgaard M Abbott NJ All vertebrates started out with a glial blood-brain barrier 4-500 million years ago Glia 2008 56 699 708 10.1002/glia.20642 18338790

[B9] Cserr HF Bundgaard M Blood-brain interfaces in vertebrates: a comparative approach Am J Physiol 1984 246 R277 88 6367490 10.1152/ajpregu.1984.246.3.R277

[B10] Thisse C Zon LI Organogenesis--heart and blood formation from the zebrafish point of view Science 2002 295 457 62 10.1126/science.1063654 11799232

[B11] Lawson ND Weinstein BM Arteries and veins: making a difference with zebrafish Nat Rev Genet 2002 3 674 82 10.1038/nrg888 12209142

[B12] Jeong JY Kwon HB Ahn JC Kang D Kwon SH Park JA Kim KW Functional and developmental analysis of the blood-brain barrier in zebrafish Brain Res Bull 2008 75 619 28 10.1016/j.brainresbull.2007.10.043 18355638

[B13] Harhaj NS Felinski EA Wolpert EB Sundstrom JM Gardner TW Antonetti DA VEGF activation of protein kinase C stimulates occludin phosphorylation and contributes to endothelial permeability Invest Ophthalmol Vis Sci 2006 47 5106 15 10.1167/iovs.06-0322 17065532

[B14] Willis CL Leach L Clarke GJ Nolan CC Ray DE Reversible disruption of tight junction complexes in the rat blood-brain barrier, following transitory focal astrocyte loss Glia 2004 48 1 13 10.1002/glia.20049 15326610

[B15] Witt KA Mark KS Hom S Davis TP Effects of hypoxia-reoxygenation on rat blood-brain barrier permeability and tight junctional protein expression Am J Physiol Heart Circ Physiol 2003 285 H2820 31 12907427 10.1152/ajpheart.00589.2003

[B16] Koto T Takubo K Ishida S Shinoda H Inoue M Tsubota K Okada Y Ikeda E Hypoxia disrupts the barrier function of neural blood vessels through changes in the expression of claudin-5 in endothelial cells Am J Pathol 2007 170 1389 97 10.2353/ajpath.2007.060693 17392177 PMC1829471

[B17] Fischer S Wobben M Marti HH Renz D Schaper W Hypoxia-induced hyperpermeability in brain microvessel endothelial cells involves VEGF-mediated changes in the expression of zonula occludens-1 Microvasc Res 2002 63 70 80 10.1006/mvre.2001.2367 11749074

[B18] Musch MW Walsh-Reitz MM Chang EB Roles of ZO-1, occludin, and actin in oxidant-induced barrier disruption Am J Physiol Gastrointest Liver Physiol 2006 290 G222 31 10.1152/ajpgi.00301.2005 16239402

[B19] Esser S Lampugnani MG Corada M Dejana E Risau W Vascular endothelial growth factor induces VE-cadherin tyrosine phosphorylation in endothelial cells J Cell Sci 1998 111 1853 65 9625748 10.1242/jcs.111.13.1853

[B20] Kevil CG Payne DK Mire E Alexander JS Vascular permeability factor/vascular endothelial cell growth factor-mediated permeability occurs through disorganization of endothelial junctional proteins J Biol Chem 1998 273 15099 103 10.1074/jbc.273.24.15099 9614120

[B21] Navaratna D McGuire PG Menicucci G Das A Proteolytic degradation of VE-cadherin alters the blood-retinal barrier in diabetes Diabetes 2007 56 2380 7 10.2337/db06-1694 17536065

[B22] Chi NC Shaw RM De Val S Kang G Jan LY Black BL Stainier DY Foxn4 directly regulates tbx2b expression and atrioventricular canal formation Genes Dev 2008 22 734 9 10.1101/gad.1629408 18347092 PMC2275426

[B23] Jin SW Beis D Mitchell T Chen JN Stainier DY Cellular and molecular analyses of vascular tube and lumen formation in zebrafish Development 2005 132 5199 209 10.1242/dev.02087 16251212

[B24] Isogai S Horiguchi M Weinstein BM The vascular anatomy of the developing zebrafish: an atlas of embryonic and early larval development Dev Biol 2001 230 278 301 10.1006/dbio.2000.9995 11161578

[B25] Her GM Chiang CC Chen WY Wu JL In vivo studies of liver-type fatty acid binding protein (L-FABP) gene expression in liver of transgenic zebrafish (Danio rerio) FEBS Lett 2003 538 125 33 10.1016/S0014-5793(03)00157-1 12633865

[B26] Abdouh M Talbot S Couture R Hassessian HM Retinal plasma extravasation in streptozotocin-diabetic rats mediated by kinin B(1) and B(2) receptors Br J Pharmacol 2008 154 136 43 10.1038/bjp.2008.48 18311190 PMC2438974

[B27] Gao BB Clermont A Rook S Fonda SJ Srinivasan VJ Wojtkowski M Fujimoto JG Avery RL Arrigg PG Bursell SE Extracellular carbonic anhydrase mediates hemorrhagic retinal and cerebral vascular permeability through prekallikrein activation Nat Med 2007 13 181 8 10.1038/nm1534 17259996

[B28] Phipps JA Clermont AC Sinha S Chilcote TJ Bursell SE Feener EP Plasma kallikrein mediates angiotensin II type 1 receptor-stimulated retinal vascular permeability Hypertension 2009 53 175 81 10.1161/HYPERTENSIONAHA.108.117663 19124682 PMC4978181

[B29] Huber JD Egleton RD Davis TP Molecular physiology and pathophysiology of tight junctions in the blood-brain barrier Trends Neurosci 2001 24 719 25 10.1016/S0166-2236(00)02004-X 11718877

[B30] Saunders NR Habgood MD Dziegielewska KM Barrier mechanisms in the brain, I. Adult brain Clin Exp Pharmacol Physiol 1999 26 11 9 10.1046/j.1440-1681.1999.02986.x 10027064

[B31] Saunders NR Habgood MD Dziegielewska KM Barrier mechanisms in the brain, II. Immature brain Clin Exp Pharmacol Physiol 1999 26 85 91 10.1046/j.1440-1681.1999.02987.x 10065326

[B32] Stonestreet BS Patlak CS Pettigrew KD Reilly CB Cserr HF Ontogeny of blood-brain barrier function in ovine fetuses, lambs, and adults Am J Physiol 1996 271 R1594 601 8997357 10.1152/ajpregu.1996.271.6.R1594

[B33] Tuor UI Simone C Bascaramurty S Local blood-brain barrier in the newborn rabbit: postnatal changes in alpha-aminoisobutyric acid transfer within medulla, cortex, and selected brain areas J Neurochem 1992 59 999 1007 10.1111/j.1471-4159.1992.tb08341.x 1494922

[B34] Mollgard K Dziegielewska KM Saunders NR Zakut H Soreq H Synthesis and localization of plasma proteins in the developing human brain. Integrity of the fetal blood-brain barrier to endogenous proteins of hepatic origin Dev Biol 1988 128 207 21 10.1016/0012-1606(88)90283-7 3289986

[B35] Daneman R Agalliu D Zhou L Kuhnert F Kuo CJ Barres BA Wnt/beta-catenin signaling is required for CNS, but not non-CNS, angiogenesis Proc Natl Acad Sci USA 2009 106 641 6 10.1073/pnas.0805165106 19129494 PMC2626756

[B36] Lee SW Kim WJ Choi YK Song HS Son MJ Gelman IH Kim YJ Kim KW SSeCKS regulates angiogenesis and tight junction formation in blood-brain barrier Nat Med 2003 9 900 6 10.1038/nm889 12808449

[B37] Liebner S Corada M Bangsow T Babbage J Taddei A Czupalla CJ Reis M Felici A Wolburg H Fruttiger M Wnt/beta-catenin signaling controls development of the blood-brain barrier J Cell Biol 2008 183 409 17 10.1083/jcb.200806024 18955553 PMC2575783

[B38] Stenman JM Rajagopal J Carroll TJ Ishibashi M McMahon J McMahon AP Canonical Wnt signaling regulates organ-specific assembly and differentiation of CNS vasculature Science 2008 322 1247 50 10.1126/science.1164594 19023080

[B39] Nitta T Hata M Gotoh S Seo Y Sasaki H Hashimoto N Furuse M Tsukita S Size-selective loosening of the blood-brain barrier in claudin-5-deficient mice J Cell Biol 2003 161 653 60 10.1083/jcb.200302070 12743111 PMC2172943

[B40] Pardridge WM Brain drug development and brain drug targeting Pharm Res 2007 24 1729 32 10.1007/s11095-007-9387-0 17629776

[B41] Abbott NJ Astrocyte-endothelial interactions and blood-brain barrier permeability J Anat 2002 200 629 38 10.1046/j.1469-7580.2002.00064.x 12162730 PMC1570746

[B42] Fadool JM Dowling JE Zebrafish: a model system for the study of eye genetics Prog Retin Eye Res 2008 27 89 110 10.1016/j.preteyeres.2007.08.002 17962065 PMC2271117

[B43] Alvarez Y Cederlund ML Cottell DC Bill BR Ekker SC Torres-Vazquez J Weinstein BM Hyde DR Vihtelic TS Kennedy BN Genetic determinants of hyaloid and retinal vasculature in zebrafish BMC Dev Biol 2007 7 114 10.1186/1471-213X-7-114 17937808 PMC2169232

[B44] Santoro MM Pesce G Stainier DY Characterization of vascular mural cells during zebrafish development Mech Dev 2009 126 638 49 10.1016/j.mod.2009.06.1080 19539756 PMC2732398

[B45] Regoli D Barabe J Pharmacology of bradykinin and related kinins Pharmacol Rev 1980 32 1 46 7015371

[B46] Bromee T Venkatesh B Brenner S Postlethwait JH Yan YL Larhammar D Uneven evolutionary rates of bradykinin B1 and B2 receptors in vertebrate lineages Gene 2006 373 100 8 10.1016/j.gene.2006.01.017 16530355

[B47] Duner T Conlon JM Kukkonen JP Akerman KE Yan YL Postlethwait JH Larhammar D Cloning, structural characterization and functional expression of a zebrafish bradykinin B2-related receptor Biochem J 2002 364 817 24 10.1042/BJ20011201 12049646 PMC1222631

[B48] Bromee T Kukkonen JP Andersson P Conlon JM Larhammar D Pharmacological characterization of ligand-receptor interactions at the zebrafish bradykinin receptor Br J Pharmacol 2005 144 11 6 10.1038/sj.bjp.0706032 15644864 PMC1575979

[B49] Westerfield M (ed) The Zebrafish Book 2007 5 Eugene, Oregon: University of Oregon Press

[B50] Xie J Fisher S Twisted gastrulation enhances BMP signaling through chordin dependent and independent mechanisms Development 2005 132 383 91 10.1242/dev.01577 15604098

[B51] Kawakami K Tol2: a versatile gene transfer vector in vertebrates Genome Biol 2007 8 Suppl 1 S7 10.1186/gb-2007-8-s1-s7 18047699 PMC2106836

[B52] Kawakami K Transgenesis and gene trap methods in zebrafish by using the Tol2 transposable element Methods Cell Biol 2004 77 201 22 full_text 15602913 10.1016/s0091-679x(04)77011-9

