# Supplementary figures and images for: A novel transgenic zebrafish model for blood-brain and blood-retinal barrier development
Source: BMC Dev Biol. 2010 Jul 23;10:76. doi: 10.1186/1471-213X-10-76 (PMC2914679; doi:10.1186/1471-213X-10-76)

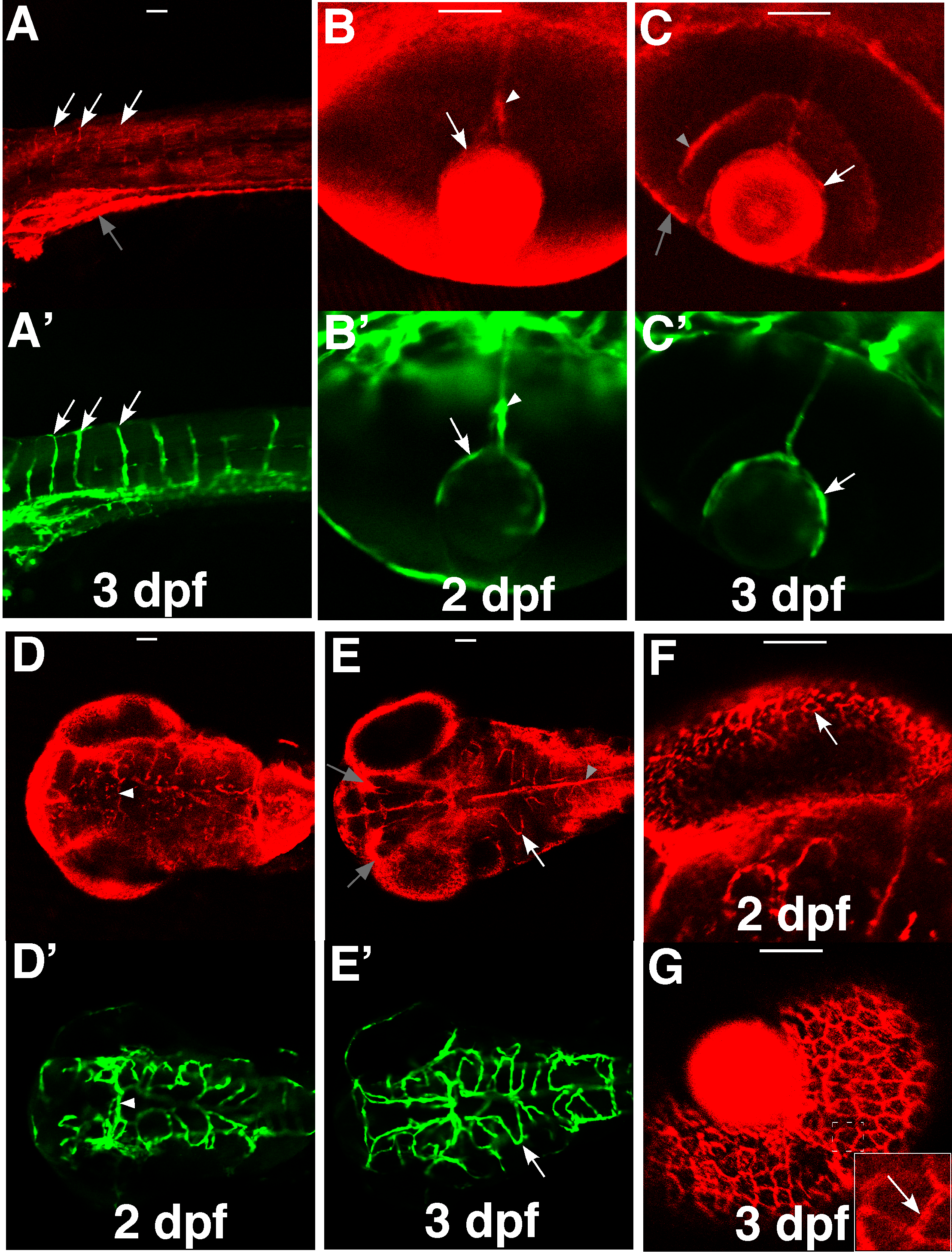

Supplement: Additional file 1 — Expression of ZO-1 in the developing BRB and BBB of zebrafish. Tg(flk1:EGFP) embryos at 2 dpf to 3 dpf were stained with rabbit anti-ZO-1; confocal images were analyzed for ZO-1 expression (red) (A-G; Alexa Fluor 568) and blood vessels (green) (A'-E'; EGFP). A&A', lateral views; the other panels, dorsal views. (A&A') The ZO-1 signal is high in the gut (shaded arrow) and low in the intersegmental vessels (arrows). (B&B') At 2 dpf, ZO-1 is localized to the HV (arrows) and the HA (arrowheads). (C&C') At 3 dpf, the HV (arrows), inner plexiform layer (shaded arrowhead) as well as the outer limiting membrane (shaded arrow) show a strong signal of ZO-1. (D&D', E&E') At 2 dpf, most brain vessels express ZO-1, including the BCA (arrowheads). Similar to the claudin-5 antibody, the ZO-1 antibody binds to many non-endothelial structures in the brain (shaded arrows and shaded arrowhead) besides the brain vasculature (arrows). (F&G) At 2 dpf, the ZO-1 antibody can stain the polygonal RPE cells (arrows) clearly. The insert in G is an enlarged view of the dashed square. Scale bars: 50 um. [file 1471-213X-10-76-S1.TIFF]

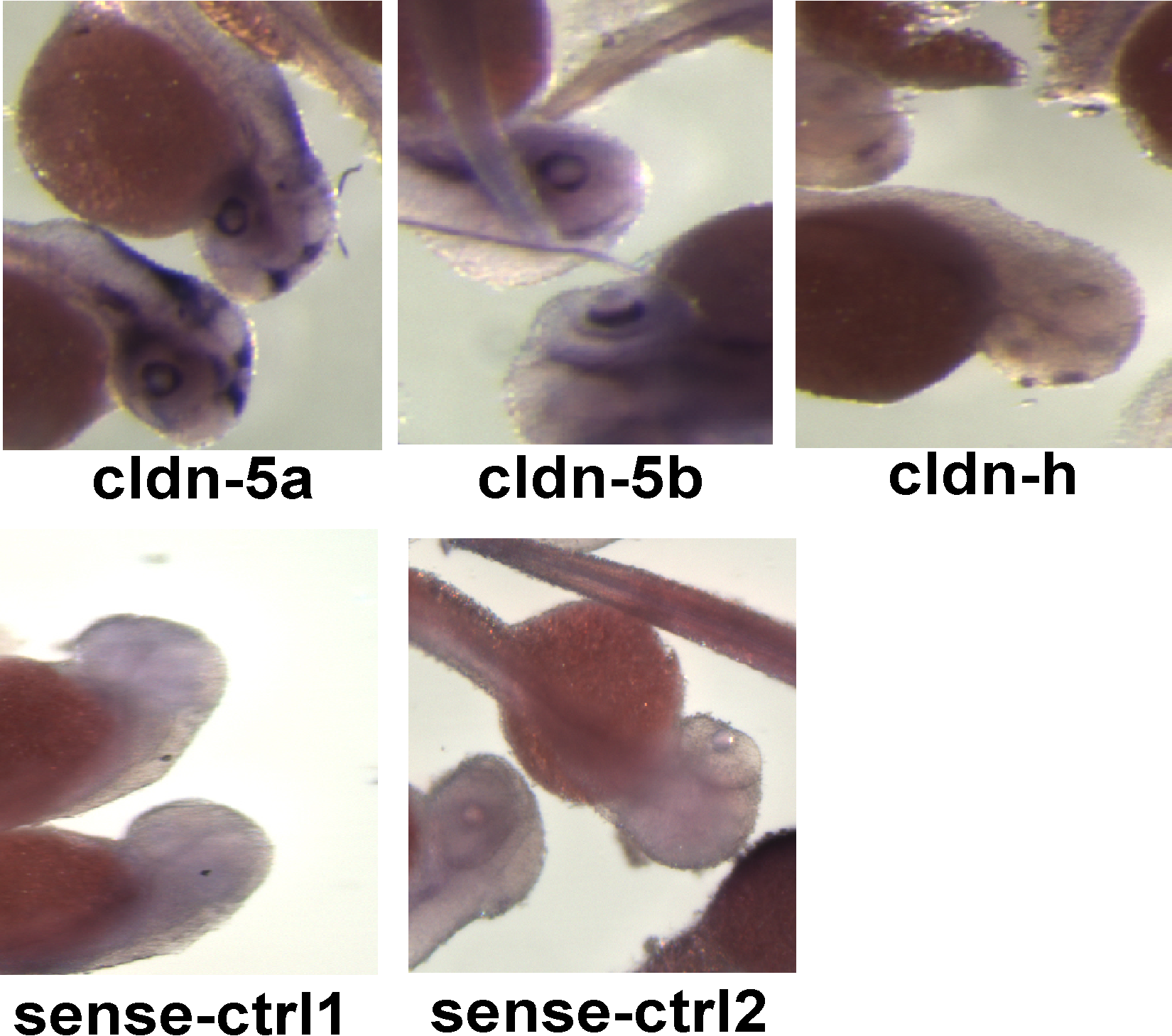

Supplement: Additional file 2 — In situ hybridization of cldn-5a, cldn-5b and cldn-h. Wild type embryos at 2.5 dpf were probed with anti-sense probes of cldn5a, cldn5b and cldn5h. The cldn-5a and cldn-5b had very strong signal in the hyaloid vasculature around the lens, while the cldn-h did not. Two sense controls did not show any signal. [file 1471-213X-10-76-S2.TIFF]

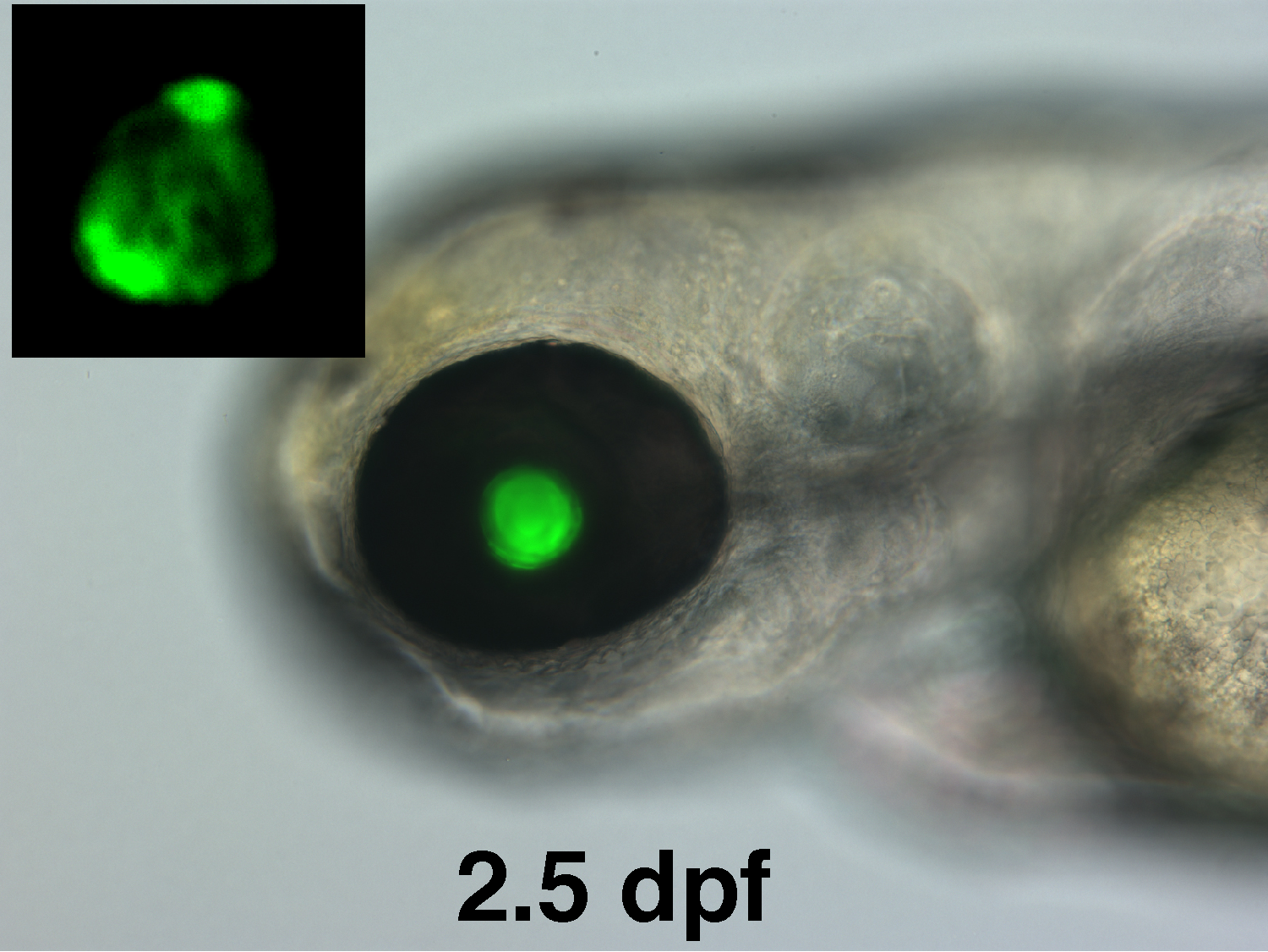

Supplement: Additional file 3 — Claudin 5b is expressed in hyaloid vessels. A 5.6 kb upstream sequence of claudin-5b can drive expression of EGFP in the hyaloid vessels. The inset is an enlarged view of the green hyaloid vessels. [file 1471-213X-10-76-S3.TIFF]

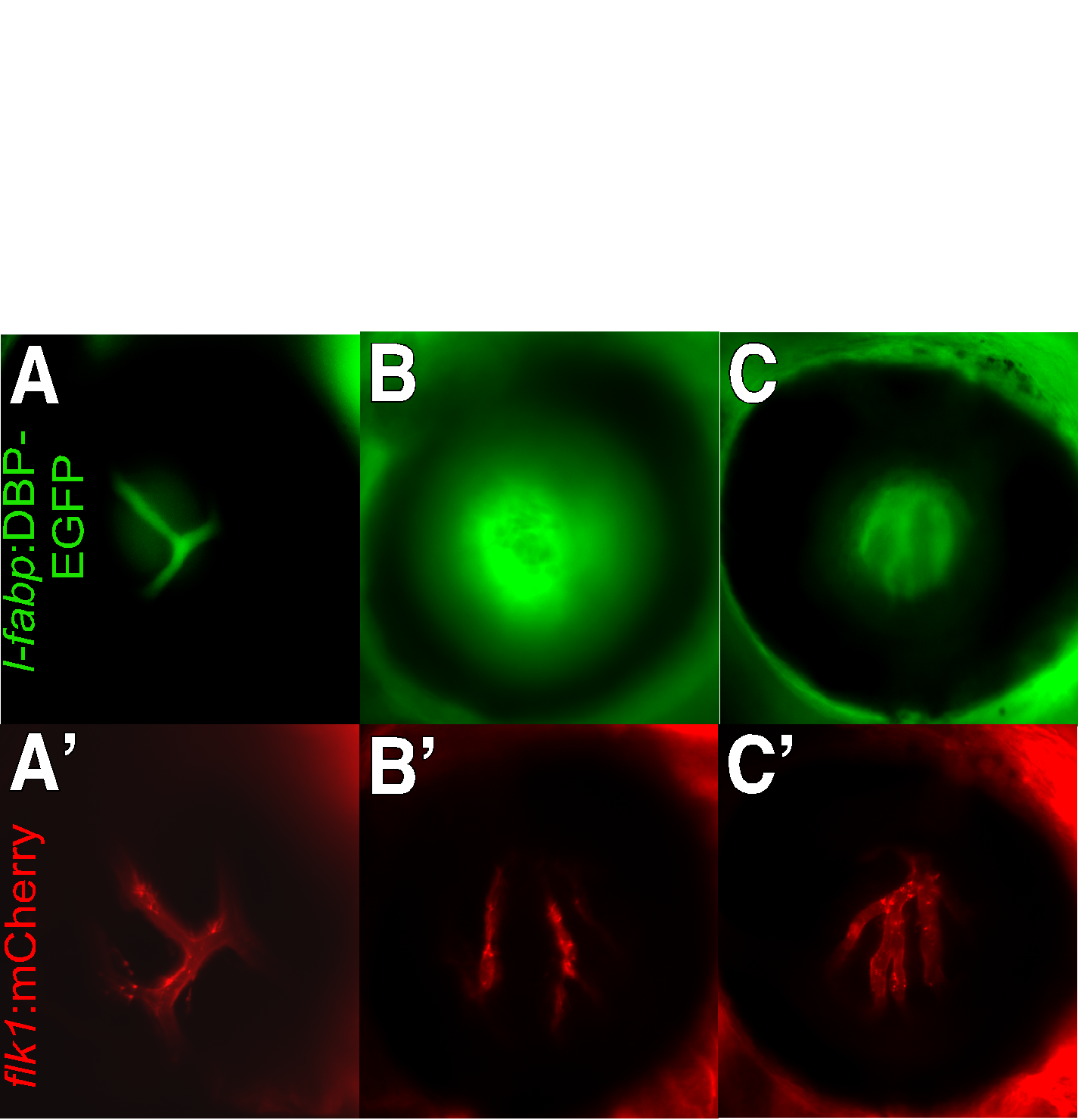

Supplement: Additional file 4 — Bradykinin induces BRB breakdown. At 5 dpf, 20 double transgenic (l-fabp:DBP-EGFP;flk1:mCherry) larvae were injected with 2 uL of 20 uM bradykinin in the right eye (B, B', C and C'). 3 hours after injection, the BRB was found to be disrupted in 18 injected eyes, as indicated by the leakage of DBP-EGFP (green) (B, C) from the hyaloid vessels (red) (B'C') which showed normal morphology. No leakage was observed in any of the BSA injected contra-lateral negative control eyes (A, A'). [file 1471-213X-10-76-S4.TIFF]

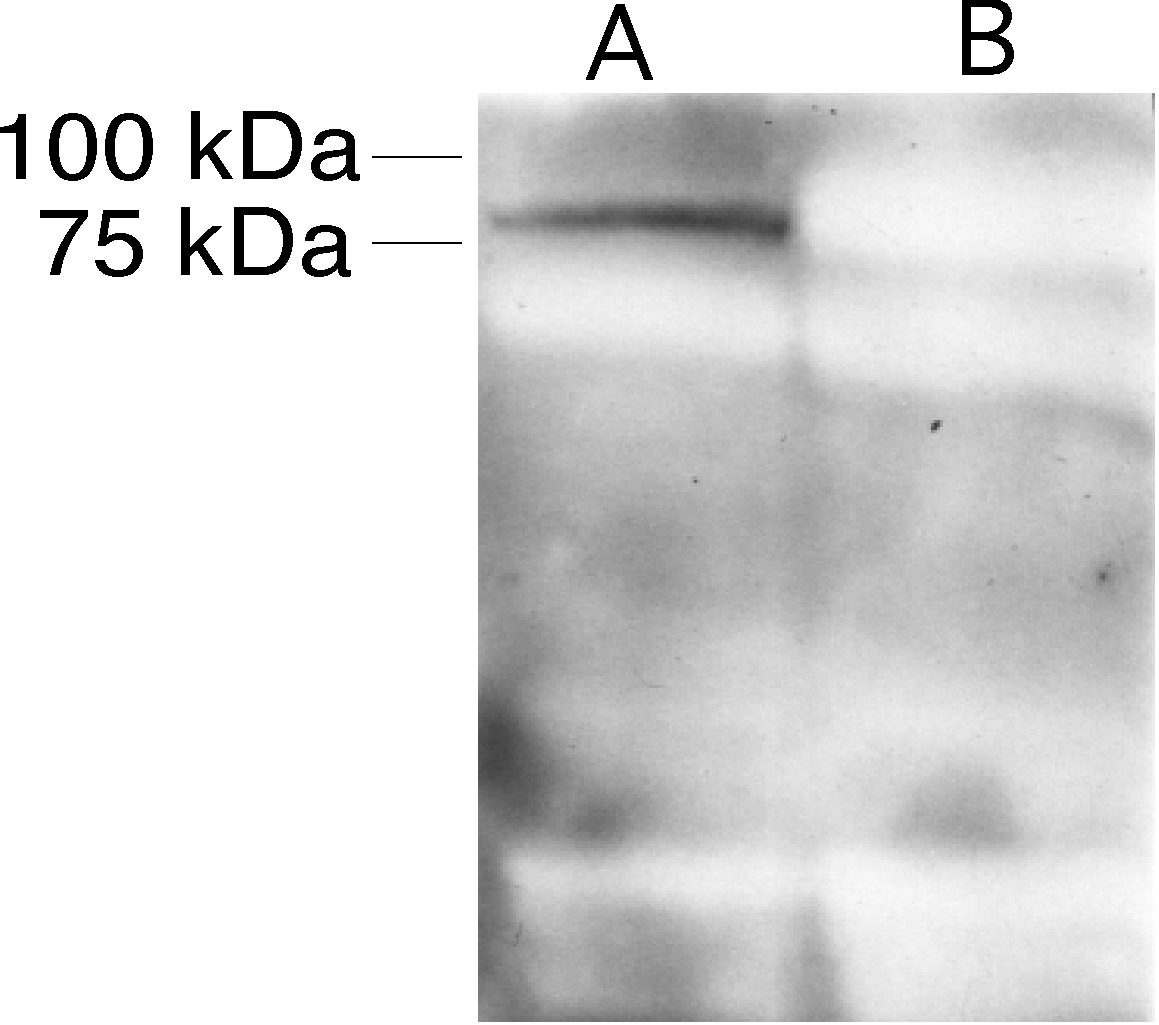

Supplement: Additional file 5 — DBP-EFGP is a 78 kDa protein in l-fabp:DBP-EGFP zebrafish. The expression level and molecular weight of DBP-EGFP was analyzed by western blot analysis of lysates from l-fabp:DBP-EGFP (A) and wild type (B) embryos using anti-GFP antibodies (Abcam, ab290). A 78 kDa protein was detected in lysates from transgenic embryos. [file 1471-213X-10-76-S5.TIFF]

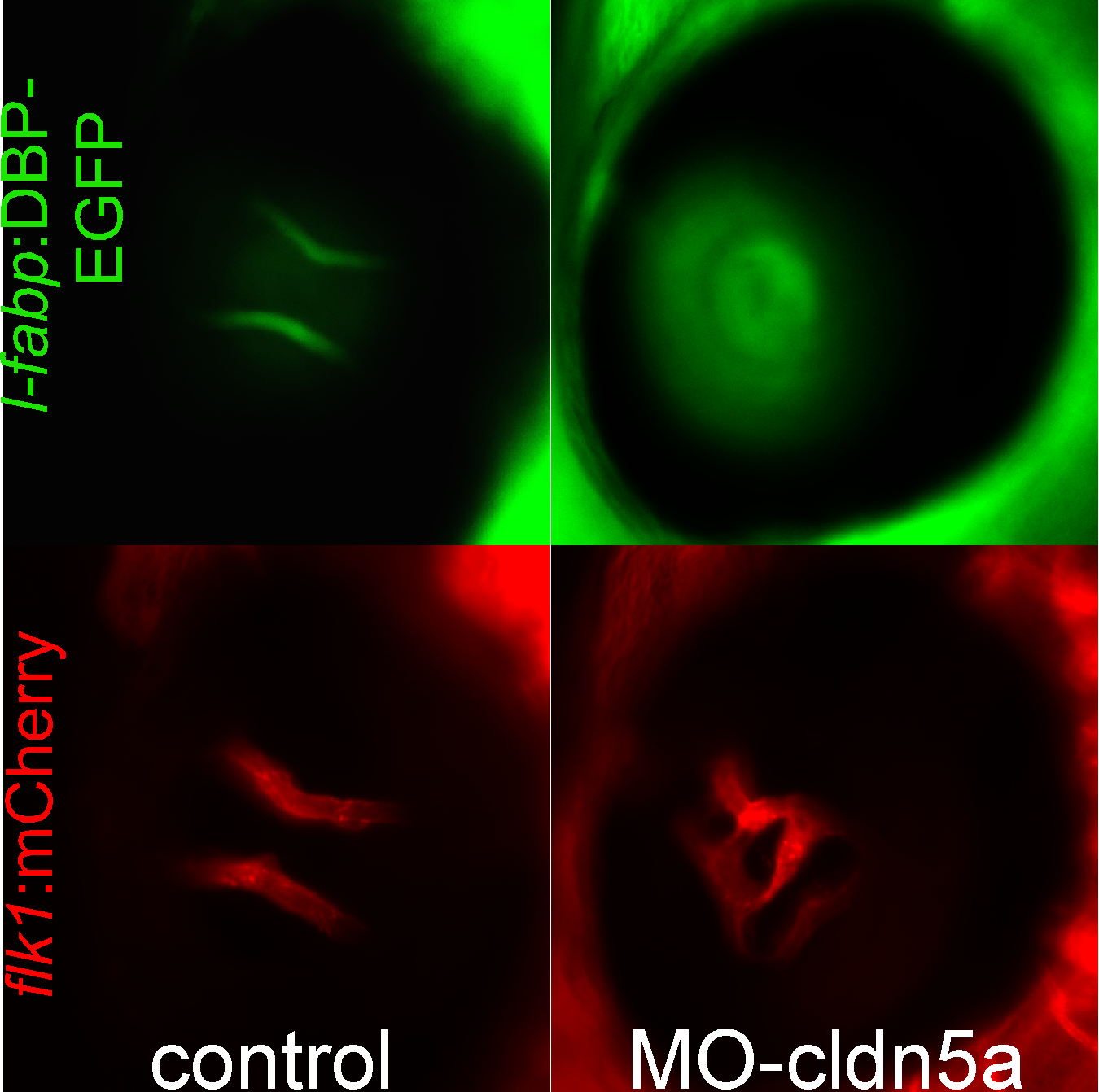

Supplement: Additional file 6 — Morpholino knockdown of claudin 5a in zebrafish embryos results in BRB breakdown. Cldn-5a was knocked down by injecting morpholino-cldn5a into double transgenic l-fabp:DBP-EGFP;flk1:mCherry embryos at the 2-cell stage. At 4 dpf, leakage of DBP-EGFP(green) from the hyaloid vasculature (red) was observed. A 5 bp-mismatched Morpholino was injected as control and showed an intact BRB. [file 1471-213X-10-76-S6.TIFF]
